# Revisiting the Ofatumumab Epitope on CD20 through Integrative Molecular Dynamics and Flow Cytometry Analyses

**DOI:** 10.34133/csbj.0096

**Published:** 2026-05-25

**Authors:** Ana Virgínia F. Guimarães, José Samuel S. Barbosa, Ana Júlia F. Lima, Marcus Rafael L. Bezerra, Daniel P. Pinheiro, Larissa Q. Pontes, Gilvan P. Furtado, Marcos R. Lourenzoni

**Affiliations:** ^1^ Protein Engineering and Health Solutions Group, Oswaldo Cruz Foundation, Fiocruz Ceará, Eusébio, Ceará, 61.773-270, Brazil.; ^2^ Postgraduate Program in Biosciences and Biotechnology, Carlos Chagas Institute/Fiocruz Paraná, Cidade Industrial de Curitiba, Curitiba, Paraná 81310-020, Brazil.; ^3^ Postgraduate Program in Biotechnology of Natural Resources, Federal University of Ceará, Campus do Pici, 825, Fortaleza, Ceará 60.440-970, Brazil.

## Abstract

Ofatumumab is a fully human anti-CD20 antibody used in B-cell malignancies and autoimmune disorders, yet its precise epitope recognition remains incompletely defined. Here, molecular dynamics simulations, binding free-energy analyses, and flow cytometry were combined to elucidate the structural basis of CD20 binding. The single-chain variable fragment recognizes a membrane-proximal epitope primarily centered on extracellular loop 2b/extracellular helix 2, stabilized by hydrophobic and electrostatic interactions involving the Y105–Y107–Y169 cluster and Arg228, while mutations at Asp99 and Tyr107 disrupted paratope stability and markedly reduced cellular binding. These results refine the molecular model of ofatumumab single-chain variable fragment–CD20 recognition and provide atomic-level insights that may assist the design of next-generation anti-CD20 and chimeric antigen receptor T-cell therapeutics.

## Highlights

• How does ofatumumab recognize CD20? Revisiting conflicting epitope models from Teeling, Du, and Kumar
• Integrative approach combining molecular dynamics, free-energy decomposition, and flow cytometry validation
• MD and Δ*G*_res analyses define an ECL2b-centered epitope consistent with cryo-EM observations by Kumar *et al.*
• Residues Tyr107, Asp99, Tyr169, and Arg228 form a stabilizing hydrophobic–electrostatic cluster in the paratope.
• Findings reconcile Teeling’s dual-loop model with modern cryo-EM data, providing insights for scFv affinity tuning in CAR-T design.

## Graphical Abstract

**Figure:**
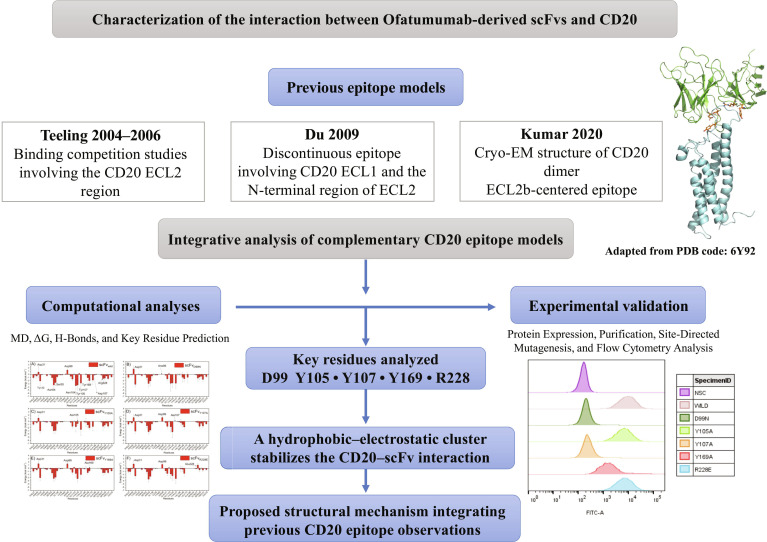

## Introduction

Monoclonal antibodies (mAbs) of the immunoglobulin G (IgG) class have profoundly shaped modern medicine, thanks to their high molecular specificity, extended serum half-life through neonatal Fc receptor recycling, and versatile effector mechanisms, including complement-dependent cytotoxicity and antibody-dependent cellular cytotoxicity [1–3]. Among therapeutic targets, CD20 stands out as a nonglycosylated phosphoprotein (33 to 35 kDa) featuring 4 transmembrane helices and 2 extracellular loops (ECL1 and ECL2). It is expressed from the pre-B to mature B-cell stages but is absent in hematopoietic stem cells and plasma cells [4–6].

Ofatumumab, a fully human IgG1κ mAb generated via transgenic mice bearing human immunoglobulin loci, shows low immunogenicity due to the complete absence of murine sequences [7–9]. Ofatumumab was initially approved by the U.S. Food and Drug Administration in 2009 for fludarabine-refractory chronic lymphocytic leukemia, and conditionally by the European Medicines Agency in 2010 for relapsed chronic lymphocytic leukemia [10–13]. It was later adopted as a monthly subcutaneous therapy for relapsing–remitting multiple sclerosis [14–17]. Beyond these indications, clinical trials have also investigated its use in rheumatoid arthritis [8,18,19], relapsed follicular lymphoma [11], and even pemphigus vulgaris [20–23], underscoring its therapeutic versatility. Despite its success, ofatumumab therapy is not without challenges. CD20 antigenic modulation, heterogeneous expression across B-cell malignancies, and reduced antibody-dependent cellular cytotoxicity under low-antigen conditions all pose limitations [4,24–26]. Furthermore, systemic administration of full-length IgG may lead to off-target Fcγ receptor engagement and infusion-related adverse events, prompting interest in engineered antibody fragments and chimeric antigen receptor T-cell (CAR-T) strategies aimed at improving specificity and safety [27–32].

At the molecular level, ofatumumab has been proposed to recognize a membrane-proximal conformational epitope on CD20. Computational prediction of B-cell epitopes remains challenging, as current methods still show limited predictive performance and highlight the need for improved structural and physicochemical representations of antigen–antibody interactions and a better understanding of key molecular features governing antibody–antigen interfaces [33,34]. Early studies based on binding competition assays and structural analyses suggested a dual-loop binding model involving residues from the small extracellular loop (ECL1) and the N-terminal region of the large extracellular loop (ECL2a) [7,35–37], in which epitope mapping was primarily achieved through x-ray crystallography of Fab–CD20 peptide complexes and mutagenesis combined with peptide scanning approaches. More recently, cryo-electron microscopy (cryo-EM) structural studies of dimeric CD20 proposed an alternative model centered on residues within the ECL2b region (Tyr161, Asn166, Glu168, Ala170, and Asn171) [38], based on cryo-EM structures of full-length CD20 in complex with therapeutic antibodies. These differing structural interpretations highlight an unresolved question regarding the precise molecular determinants of ofatumumab recognition. To address this gap, we combined atomistic molecular dynamics (MD) simulations, binding free-energy calculations, and site-directed mutagenesis validated by flow cytometry to structurally characterize and refine the functional epitope of the ofatumumab single-chain variable fragment (scFv) on CD20, within the context of previously proposed epitope models, allowing the evaluation of residue-level contributions and conformational dynamics in a membrane-embedded environment, which are not fully captured by static structural approaches. We focused on the scFv format, which links the variable heavy (VH) and variable light (VL) domains via a flexible linker, offering a smaller and tractable molecular system for simulation and expression studies while preserving antigen-binding capability [28,39–41].

By reconciling the previous epitope models described by Teeling *et al.* and Kumar *et al.* [7,38], this study defines the structural determinants underlying ofatumumab scFv–CD20 stability, offering a basis for rational optimization of binding. Notably, ofatumumab is a fully human antibody, in contrast to rituximab-derived antibodies, which are chimeric murine–human molecules, providing additional motivation to investigate the structural features of fully human anti-CD20 binders. This is particularly relevant for CAR-T design, where stronger binding does not necessarily lead to improved therapeutic performance [42,43] as CAR-T functionality is influenced by multiple additional parameters, including scFv structural organization, spacer architecture, and intracellular signaling domains.

## Materials and Methods

### Construction of scFvs from ofatumumab Fab

The 3-dimensional structures of VH and VL domains used for the assembly of the native scFv, named here as scFv_wild_ (sequence shown in Fig. S1), were obtained from the atomic coordinates of the ofatumumab Fab structure available at Protein Data Bank (PDB ID: 6Y92 - full-length CD20 in complex with ofatumumab Fab). A (GGGGS)_3_ linker structure was modeled using Modeller version 10.4 [44] to connect the variable domains to form the scFv. The linker was positioned to connect the C-terminus of VH to the N-terminus of VL. The homology modeling was carried out through the missing residues protocol, in which 250 models were generated, and the best one was chosen based on thediscrete optimized protein energy score [45]. The best complete scFv 3D structure of scFv_wild_ obtained was subjected to MD simulation in complex with CD20, as detailed in the subsequent sections, and then was later used to model the scFv variants. Each mutation was modeled using the PyMol software version 3.1.3 (Schrödinger, LLC) through the mutagenesis option. Five structures containing single-point mutations at strategic positions were generated to assess the importance of each residue in maintaining the interface with CD20. These variant structures were named according to the amino acid substitution and its position in the sequence scFv_type_, type: D99N, Y105A, Y107A, Y169A, and R228E (Figs. S1 and S2).

### Preparation of systems with CD20 in interface with scFvs

The human CD20 3D structure was obtained from the PDB (PDB ID: 6Y92), and the amino acid sequence used corresponded to the CD20 human reference sequence obtained from UniProt (accession P11836, CD20_HUMAN) excluding the N- and C-terminal regions (Fig. S3). The scFv_wild_ modeled structure was superimposed using PyMOL software version 3.1.3 (Schrödinger, LLC) onto the cryo-EM structure of the ofatumumab Fab (PDB ID: 6Y92), to correctly position the scFv for interaction with CD20. The resulting root-mean-square deviation (RMSD) was close to zero, as the VH and VL regions of the scFv were directly extracted from the Fab structure. The scFv_wild_ was then saved together with CD20 in the same file (scFv_wild_/CD20), preserving the coordinates of the interaction interface between the molecules. The scFv_wild_/CD20 complex was subjected to a 700-ns MD simulation, as described below. The final structure from this simulation was then used as a template to construct the systems of the scFv variants with CD20.

From this final configuration of the scFv_wild_/CD20 system (700 ns), point mutations corresponding to each scFv variant were introduced directly in PyMOL (version 3.1.3, Schrödinger, LLC), generating the scFv_variant_/CD20 complexes while preserving the structural context of the interaction interface. This approach preserved the spatial coordinates of the interaction interface between the scFvs and CD20. Each complex of scFv_type_/CD20 was subsequently saved in a single structure file, and systems were solvated. The same final configuration of the scFv_wild_/CD20 system (700 ns) was also used as the structural reference to generate the isolated scFv_wild_ and scFv_variant_ systems in aqueous solution, allowing the evaluation of mutation-induced structural changes in the scFvs independently of the preformed antigen interface.

### Insertion of scFv/CD20 in a lipid bilayer

The structure of the scFv_wild_ in complex with CD20 (scFv_wild/_CD20) was submitted to the CHARMM-GUI program [46–48] to be inserted in a lipid bilayer with the same lipid composition as the human B-lymphocyte membrane [49]. The membrane model, measuring 12 × 12 nm, is composed of 512 lipid molecules, including phosphatidylcholine and phosphatidylethanolamine (PE) in a ratio of 0.64 and 1.72, respectively. Phosphatidylserine and phosphatidylinositol were excluded due to their low abundance relative to phosphatidylcholine and PE and because their negative charge could introduce simulation artifacts and increase computational cost. Since the CD20 structure used to build the scFv_type_/CD20 systems was obtained from the final structure of the scFv_wild_/CD20 simulation, the membrane used for assembling the variant systems was the same as that described for the native complex. Thus, the same membrane with the anchored CD20 was used in all systems.

### MD simulation

MD simulations were carried out using GROMACS version 2024.1 [50,51] with the CHARMM36 force field [52]. Two types of systems were simulated: (a) scFv interacting with the extracellular CD20 domain embedded in a lipid bilayer model representing the B-lymphocyte membrane (scFv_type_/CD20) and (b) isolated scFv in aqueous solution (scFv_type_), allowing the evaluation of intrinsic conformational stability independently of the antigen interface. The term “type” denotes either the wild-type scFv or the mutant variants D99N, Y105A, Y107A, Y169A, and R228E.

Protonation states were assigned using H++ server [53–55], considering the pH of 7.4 (physiological pH). The scFv/CD20 complex embedded in the lipid bilayer was surrounded by a water layer TIP3P model [56] on the upper and lower faces relative to the z-axis of the simulation box, scaled to dimensions of 13 × 13 nm (xy) × 16 nm (z). For the isolated scFvs, dodecahedral boxes were used with a minimum distance of 1.8 nm between the protein and the box edges, using the same solvent model. The complete atomic compositions of the systems are shown in Table S1. Protein bond lengths were maintained using the LINCS (Linear Constraint Solver) algorithm [57,58], while water geometry was preserved with SETTLE [59]. Nonbonded interactions, including van der Waals and Coulomb forces, were calculated using a 1.2-nm cutoff, and long-range electrostatics were treated with the particle mesh Ewald method [60].

To optimize the geometry of the systems, distinct energy minimization protocols were employed depending on system composition. For the membrane-embedded scFv_type_/CD20 complexes, the systems underwent a 3-step energy minimization protocol. The first and third steps used the steepest descent method, while the second employed the conjugate gradient algorithm. Throughout this process, positional restraints were applied to the protein and membrane to maintain structural stability. For isolated scFv_type_ systems in aqueous solution, energy minimization was performed using a single-step steepest descent algorithm.

To achieve a physiological ionic concentration of 0.15 M, Na^+^ and Cl^−^ ions were added to neutralize the systems, which were then subjected to thermalization steps in the canonical (NVT) and isothermal–isobaric (NPT) ensembles (Table S2). A detailed description of the thermalization protocol is provided in Table S2. During these stages, positional restraints were applied to the protein and membrane (when applicable), with a final unrestrained NPT step performed prior to production. The temperature was controlled by the V-rescale thermostat [61], while the system pressure was controlled with the C-rescale [62] in thermalization, and Parrinello–Rahman [63,64] in trajectory acquisition. Pressure was controlled at 1 atm, at a temperature of 310 K. The integrator used to solve the equations of motion was the leap-frog algorithm [65]. Trajectory acquisition phase was carried out in the NPT ensemble with a dt = 2 fs, where the coordinates were recorded every 100 ps. The scFv_wild_/CD20 system was subjected to 700 ns of MD simulation, while each of the scFv_mutant-variants_/CD20 complexed with CD20 systems was simulated for 200 ns. Isolated scFv_type_ systems were simulated for 300 ns under the same conditions.

### Trajectory analysis

The intermolecular interaction potential (IIP) between scFv and CD20 was calculated as the sum of Lennard-Jones and electrostatic interaction energies between atoms of both molecules within a 1.3-nm cutoff, using the GROMACS version 2024.1 software package [50,51] and the binding free energy (Δ*G*_binding_) was evaluated using the gmx_MMPBSA program [66]. The gmx rms program was used to calculate the RMSDs of carbon α atoms (Cα) of the protein structures collected in the trajectory over time after overlapping to the MD initial structure. For each system, RMSD values were calculated relative to its own initial configuration. The variant scFv_type_/CD20 systems were generated by introducing point mutations into the reference scFv_wild_/CD20 structure obtained from the final configuration of the MD trajectory at 700 ns, ensuring preservation of the overall backbone geometry at the start of the simulations. The same reference configuration was also used to generate the corresponding scFv_type_ systems simulated in aqueous solution. Additionally, the minimum distance between Asp99 and Arg228 residues was monitored using the gmx mindist tool. The IIP was determined using the gmx energy tool by summing the long-range (electrostatic) and short-range (Lennard-Jones) potentials between the scFvs and the CD20 within a 1.3-nm cutoff radius. The Δ*G*_bind_ between the scFv and CD20 was calculated considering an ionic concentration of 0.15 M, a temperature of 298.15 K, a solvent dielectric constant of 80, a solute dielectric constant of 1, and a membrane dielectric constant of 7. Additionally, the Δ*G*_bind_ per residue was calculated within a 6-Å cutoff radius between the scFv and CD20 residues using themolecular mechanics Poisson–Boltzmann surface area (MM-PBSA) method (gmx_MMPBSA program). All plots were generated with the Origin 8.0 program.

### Bacterial strain transformation

The synthetic gene encoding the ofatumumab-derived scFv_wild_, with codon optimization for *Escherichia coli* (*E. coli*), was commercially acquired from GenScript. The gene sequence (732 bp) was initially inserted into the pUC-57 cloning vector and subsequently subcloned into pET-SUMO expression vector. The recombinant plasmid was transformed into *E. coli* Shuffle B T7 Express (New England Biolabs, 2024), a strain derived from *E. coli* Shuffle B T7 and specifically engineered to enhance disulfide bond formation in the cytoplasm, thus promoting the soluble expression of recombinant proteins.

Transformation was carried out via heat shock. Briefly, 30 ng of plasmid DNA was incubated with competent cells (50-μl aliquot of cells) on ice for 30 min, followed by exposure to 45 °C for 50 s and subsequent return to ice for 2 min. Then, 1 ml of super optimal broth with catabolite repression medium was added and incubated for 1 h at 37 °C with shaking at 200 rpm. After centrifugation at 10,000 × g for 40 s, the supernatant was discarded, and the cell pellet was resuspended in the residual medium. A 40-μl aliquot of the suspension was plated onto Luria–Bertani (LB) agar (10 g/l tryptone, 5 g/l yeast extract, 10 g/l NaCl, and 2% [m/v] agar) containing kanamycin (40 μg/ml) and incubated at 37 °C for 16 h.

### Expression of scFv in *E. coli* Shuffle T7

For recombinant expression, *E. coli* Shuffle B cells harboring the pET-SUMO-scFv constructs were cultured in LB medium (10 g/l tryptone, 5 g/l yeast extract, and 10 g/l NaCl) supplemented with kanamycin (40 μg/ml). An inoculum corresponding to 2% (v/v), prepared from a preculture grown at 37 °C for 16 h, was used to initiate the expression culture. Cells were incubated at 37 °C, 200 rpm, until reaching an optical density at 600 nm of 0.4 to 0.6.

Induction with 0.4 mM isopropyl β-d-1-thiogalactopyranoside was followed by incubation for 16 h at 24 °C with shaking at 200 rpm. Samples were collected before (*T*_0_) and after induction (*T*_16_) and analyzed by sodium dodecyl sulfate-polyacrylamide gel electrophoresis (SDS-PAGE). The cell pellet obtained after centrifugation (8,000 × g, 10 min, 4 °C) was used for protein purification.

### Protein purification, SDS-PAGE, and immunoblotting

#### Protein purification

Purification of scFv_wild_ and variants was performed via immobilized metal affinity chromatography using an ÄKTA Pure 25 system (Cytiva) and a 1-ml HisTrap HP column (Cytiva). Pellets obtained from 250 ml of cultures were resuspended in Buffer A (50 mM tris and 300 mM NaCl, pH 8.0) containing 20 mM imidazole, 1% Triton X-100, 1 mM phenylmethylsulfonyl fluoride, and a protease inhibitor cocktail (P8340, Sigma-Aldrich). Cell lysis was performed by sonication (QSonica Q700, probe 4420) using 5-s pulses alternated with 30-s rests for 10 min on ice.

After centrifugation (10,000 × g, 30 min, 4 °C), the supernatant was filtered through a 0.22-μm polyethersulfone filter and loaded onto the column pre-equilibrated with Buffer A. Elution was carried out using 100% of Buffer B (Buffer A + 500 mM imidazole). To remove imidazole and protein aggregates, the samples were subjected to size exclusion chromatography (Sephacryl S-200 HR) in phosphate-buffered saline (PBS) and then concentrated using Amicon Ultra filters (molecular weight cutoff of 10 kDa).

The SUMO tag was removed by enzymatic cleavage using ubiquitin-like-specific protease 1 (ULP-1; SUMO protease), which was previously expressed and purified by the research group. The reaction (10:1 protein: ULP-1 ratio) was carried out at 4 °C for 16 h. Cleaved proteins were recovered as flow-through fractions after a second immobilized metal affinity chromatography step and monitored by absorbance at 280 nm.

#### SDS-PAGE and immunoblot

Eluted fractions were analyzed by reducing SDS-PAGE (12.5%) and stained with Coomassie Brilliant Blue G-250. Parallel samples were transferred onto nitrocellulose membranes (Trans-Blot Turbo, Bio-Rad), blocked with 5% skim milk in phosphate-buffered saline with Tween 20 , and incubated with horseradish peroxidase-conjugated anti-hemagglutinin (HA) mAb (Invitrogen, 1:5,000 v/v). Detection was performed via chemiluminescence.

### Eukaryotic cell culture and flow cytometry

Human cell lines Raji (CD20^+^) and Jurkat (CD20^−^) were maintained in RPMI 1640 medium supplemented with 10% fetal bovine serum and 1% penicillin/streptomycin, at 37 °C under 5% CO₂.

For flow cytometry analysis, cells were stained with trypan blue for viability assessment, adjusted to 1 × 10^6^ cells/ml, and plated in round-bottom 96-well plates. Subsequently, cells were incubated with scFvs (1,000 ng, 25 °C, 30 min), washed with fluorescence-activated cell sorting buffer (PBS + 2% fetal bovine serum + 2 mM EDTA), and incubated with fluorescein isothiocyanate (FITC)-conjugated anti-HA secondary antibody (1:500 v/v). As a staining control, cells were incubated, in a parallel well, with a phycoerythrin-conjugated anti-CD20 antibody (BD Pharmingen, clone 2H7, cat. 555623) to validate CD20 expression in the cell lines. Cells were also incubated with the secondary antibody (anti-HA FITC) only as nonspecific control. The assay was performed in biological duplicate and experimental triplicate.

Analyses were performed using a FACSCelesta cytometer (BD Biosciences), acquiring 10,000 events (viable cells) per sample. Data were processed using FlowJo software, applying gating strategies to select viable cells and assessing fluorescence signals corresponding to scFv binding. Mean fluorescence intensity (MFI) values were measured and normalized with the nonspecific control to generate the relative MFI (rMFI) values.

### Work with scFv variants

Point mutations were introduced using mutagenic polymerase chain reaction (PCR), employing specific pairs of oligonucleotides for each substitution. The amplification protocol was performed in a thermal cycler under the following conditions: initial denaturation at 98 °C for 30 s, followed by 12 cycles of denaturation at 98 °C for 30 s, annealing at 60 °C for 30 s, and extension at 72 °C for 1 min [67]. Each 50-μl reaction was carried out individually to introduce a single mutation per reaction into the plasmid containing the native scFv gene. The PCR was prepared following high-fidelity Phusion polymerase protocol. Each mutagenic primer (500 nM), 0.8 nM dNTP mix, 1 U of Phusion High Fidelity Polymerase (ThermoFisher Scientific), and 20 ng of template DNA were used to perform each site-directed mutagenesis. Primer sequences designed for each mutation are listed in Table S3. PCR template was digested with *DpnI* enzyme (Promega) for 1 h at 37 °C.

## Results

### In silico results

#### Structural analysis of the scFv/CD20 complexes and scFvs in aqueous solution

We performed a long-time-scale MD simulation, 700 ns for the scFv_wild_/CD20 system and 200 ns for the variants scFv_type_/CD20 systems, to evaluate structural changes in relation to the initial reference structure, corresponding to the final configuration of the scFv_wild_/CD20 complex at 700 ns (Fig. S4A to F). The RMSD profiles indicate changes in the structures of the scFvs and CD20 throughout the simulation, as well as alterations in the relative orientation of the scFv at the CD20 interface (Fig. S4A to F). As is well known, the scFv linker region exhibits intrinsic mobility; therefore, the Cα atoms of these residues were excluded from the calculations. Including this region would result in high RMSD values, hindering the structural analysis of the VH+VL domains.

In the scFv_wild_/CD20 system (Fig. S4A), the RMSD profile of CD20 shows 2 plateaus: In the initial nanoseconds, the RMSD reaches approximately 0.2 nm and stabilizes around 0.35 nm by ~100 ns, with low oscillation amplitude. The RMSD profiles of the VH, VL, and VH+VL domains of the scFv are like that of CD20, with a sharp increase around 100 ns followed by stabilization. We defined structural equilibrium as being reached at teq = 100 ns, which was used as the starting point for average calculations. Between ~450 and 600 ns, the VH and VH+VL domains exhibited strong oscillations, although smaller in the VH+VL complex, indicating that the instability originates mainly in VH and not from changes in the VH/VL orientation. The RMSD of the N-terminal region was calculated after superposing the Cα atoms of VH, excluding the N-terminal’s own Cα atoms (Fig. S5A), with RMSD values reaching ~2 nm. The resulting profile closely resembles that observed for VH (Fig. S4A), confirming that the instability between 450 and 600 ns results from the displacement of the N-terminal region, which initially formed a β-sheet.

Structural snapshots at different time points (80, 400, 464, and 700 ns) revealed displacement of the N-terminal region (residues 1 to 13) (Fig. S7). Until ~100 ns, this region formed a β-sheet with residues 16 to 19 (Fig. S6A); subsequently, the β-sheet broke down, and the VH RMSD reached ~0.6 nm (Fig. S4A). The sharp increase in VH RMSD after 100 ns (Fig. S4A) corresponds to a shift in the N-terminal position, as confirmed by the RMSD in Fig. S5A. Between 450 and 600 ns, intense RMSD oscillations were observed, indicating increased N-terminal motion, further distancing from its original position (Fig. S6C), with rapid fluctuations reflecting its structural instability. After 600 ns (Fig. S5A), the region returned to a conformation like that at 400 ns (Fig. S6A and D), as supported by similar RMSD values, reinforcing that the final simulation configuration represents the scFv_wild_/CD20 interface. Average RMSD values were 0.29 ± 0.02 nm for VH, 0.28 ± 0.01 nm for VH+VL, 0.24 ± 0.01 nm for VL, and 0.32 ± 0.01 nm for CD20 (Table S4). These low average RMSD values and deviations indicate high structural stability of both the scFv and CD20.

To assess the possibility of relative movement of the scFv in relation to the initial structure, CD20 Cα atoms were aligned to the reference structure, and the RMSD of the native scFv was calculated (Fig. S5B). The RMSD profile shows that, during the initial nanoseconds, a change in the relative position of the scFv occurred, reaching 1.28 nm and decreasing to 0.35 nm within 25 ns. At 35 ns, a peak of 2.6 nm is observed, followed by periodic peaks up to 300 ns, suggesting that this period corresponds to the accommodation of the scFv/CD20 interface at approximately 1.2 nm (Fig. S5B). For the variant scFvs (Fig. S4B to F), RMSD analysis indicated that the structures remained stable and that the mutations did not significantly affect the structural stability of the scFvs. The RMSD profiles were lower than those observed for the scFv_wild_/CD20 complex (Table S4), with RMSD < 0.21 ± 0.01 nm calculated for the scFv_Y105A_/CD20 system. No significant changes were observed in the N-terminal position relative to the final structure of scFv_wild_/CD20, corroborating the structural stability of this region.

For the systems containing the mutant variants, the RMSD curves showed rapid structural stabilization within the first nanoseconds (Fig. S4B to F), reaching values close to ~0.2 nm or lower, compared to ~0.28 nm observed for the scFv_wild_/CD20 system (Table S4). Similarly, CD20 structure in the variant systems exhibited RMSD values below 0.15 nm, whereas in the scFv_wild_/CD20 system, this value reached ~0.32 nm. All systems showed high structural stability in the VH, VL, and VH+VL domains, except for the scFv_Y105A_/CD20, which exhibited reduced structural stability in the VL domain and, consequently, in the corresponding VH+VL profile. In this context, teq = 100 ns was selected for the calculation of average values for the variant systems, as the RMSD curves were already stabilized within this interval. This faster stabilization is consistent with the fact that the variant systems were generated from the final equilibrated configuration of the scFv_wild_/CD20 system, indicating that the structural modifications induced by the mutations occurred locally at the interface relative to the original structure.

To further investigate whether the observed structural stability within the scFv/CD20 complexes reflected intrinsic properties of the scFv variants or resulted from antigen-mediated stabilization, additional MD simulations were performed in aqueous solution (scFv_type_). RMSD analysis revealed differential mobility patterns among variants, particularly affecting the complementarity-determining region 3 of the heavy chain (CDR-H3) (Fig. S7). In the scFv_wild_, Asp99 contributes to stabilizing the internal region of CDR-H3, maintaining a relatively conserved loop orientation (Fig. S7A and G). RMSD profiles show that similar structural behavior was observed for the scFv_Y105A_, indicating limited structural perturbation relative to the wild-type scaffold (Fig. S7A and C). In contrast, the scFv_Y107A_ showed reduced Arg228–Asp99 distances (0.30 ± 0.17 nm), consistent with stable salt-bridge formation and torsional displacement of CDR-H3 (Fig. S7D and J). The scFv_Y169A_ also promoted Arg228–Asp99 proximity (0.42 ± 0.25 nm), suggesting a transient electrostatic interaction (Fig. S7E and K). Charge-altering mutations, such as D99N and R228E in scFv_D99N_ and scFv_R228E_, disrupted the electrostatic balance responsible for stabilizing CDR-H3, mediated by Asp99 and by a cation–π interaction between Tyr107 and Arg228 (Fig. S7B, F, H, and L). In the scFv_wild_, the Tyr107–Arg228 distance averaged 0.47 ± 0.09 nm over the last 200 ns, consistent with a stabilizing cation–π interaction, whereas in the scFv_Y169A_, the Tyr169–Arg228 distance increased to 0.63 ± 0.15 nm, suggesting a weaker and likely transient interaction.

#### IIP between scFvs and CD20

The IIP between the scFvs and CD20 was calculated using a cutoff radius of 1.3 nm, aiming to assess the stability of the scFv/CD20 interface and to determine whether mutations in the scFvs could affect it. The IIP represents the sum of nonbonded atomic potential contributions. In the IIP profiles (Fig. S8A to F), the values remained below 0 kcal·mol^−1^ throughout the simulation, indicating attraction between the scFvs and CD20.

In the scFv_wild_/CD20 system (Fig. S8A), the curve decreases by approximately −100 kcal·mol^−1^ until around 300 ns, reaching −220 kcal·mol^−1^. From this point onward, the IIP remains oscillating around −220.1 ± 27.5 kcal·mol^−1^. This decrease in potential supports the RMSD observations of the scFv relative to CD20, suggesting that 300 ns is the time required for interface accommodation. The reduction in IIP over time indicates a reorganization of the interface, resulting in better accommodation of the scFv to CD20 and, consequently, a more negative potential. Figure S9 illustrates the interface of the scFv_wild_/CD20 complex. Figure S9A to C shows 3 views of the initial simulation configuration, which closely resembles the cryo-EM structure deposited in the PDB (code 6Y92), while Fig. S9D to F shows views of the final MD configuration.

All systems containing variant scFvs (Fig. S8B to F) showed IIP values slightly higher than that of scFv_wild_/CD20 (−220.1 ± 27.5 kcal·mol^−1^) (Fig. S8). The substitution of a positively charged residue with a negatively charged one, as in the scFv_R228E_/CD20 system (Fig. S8F), resulted in an IIP of −175.2 ± 19.7 kcal·mol^−1^. The mutation that reduced the negative charge, such as in the scFv_D99N_/CD20 system (Fig. S8B), showed an even less negative value (−159.8 ± 28.8 kcal·mol^−1^). These results indicate that the D99N mutation in the VH domain had a greater effect on increasing the IIP than the R228E mutation in the VL domain, suggesting that the neutralization of the negative charge at Asp99 is more disruptive for the stability of the scFv/CD20 interface than the charge reversal introduced by Arg228E. The other mutations involved tyrosine residues, which are slightly polar, and were replaced by alanine residues (Fig. S8C to E). Nevertheless, all average IIP values for the variants were higher than that of the scFv_wild_/CD20, reinforcing the importance of these residues for maintaining the interface and for interactions with specific regions of CD20. Structural comparison of the initial (Fig. S9A to C) and final configurations (Fig. S9D to F) further demonstrated that the scFv changed its relative orientation on CD20 during the simulation, thereby establishing contacts with residues different from those observed in the initial cryo-EM (PDB ID: 6Y92). Principal component analysis (Fig. S9G and H) revealed a large variation in principal component 1 (PC1) and principal component 2 (PC2), with PC1 showing the greatest fluctuation until ~300 ns, after which both PC1 and PC2 stabilized for the remainder of the simulation. The PC1/PC2 projection indicated diffuse regions that converged into a predominant cluster after 300 ns, located between −100 to 0 for PC1 and −50 to 50 for PC2. This metric further confirms the observations derived from RMSD, IIP, and the structural snapshots (Fig. S9A to F), supporting that a major rearrangement occurred in the scFv/CD20 interface compared to the cryo-EM reference structure (PDB 6Y92).

#### Binding free energy and structural analysis of scFv/CD20 interactions

To further analyze the stability of the scFv/CD20 interface and the individual contribution of each residue, the binding free energy (Δ*G*_binding_) between the scFvs and CD20 was calculated using the MM-PBSA method over MD simulations, as illustrated in Fig. S10 and Table S5. The Δ*G*_binding_ profiles exhibit predominantly negative oscillations near zero throughout the simulation. As previously observed in the IIP and RMSD parameters, the scFv_wild_/CD20 interface stabilizes after 300 ns. Therefore, average Δ*G*_binding_ values were calculated over the 300- to 700-ns interval. During this period, the scFv_wild_/CD20 Δ*G*_binding_ fluctuated around −34.5 ± 15.6 kcal·mol^−1^.

For the variant scFvs, average Δ*G*_binding_ values were calculated over the 100- to 200-ns interval, as RMSD analysis demonstrated rapid stabilization of the scFv_type_/CD20 complexes within the first 100 ns due to their generation from the final equilibrated configuration of the scFv_wild_/CD20 system. The Δ*G*_binding_ values of the variant scFvs indicated that the D99N (−13.4 ± 14.5 kcal·mol^−1^) and R228E (−19.1 ± 11.3 kcal·mol^−1^) mutations caused the greatest reductions in binding affinity compared to the native scFv. The Y105A (−26.2 ± 12.1 kcal·mol^−1^) and Y169A (−24.0 ± 10.6 kcal·mol^−1^) mutations had intermediate values, while Y107A (−34.4 ± 10.9 kcal·mol^−1^) remained close to the scFv_wild_ value. Nevertheless, the high and similar SDs across systems prevent definitive claims of statistically significant differences in binding strength.

**Fig. 1.**
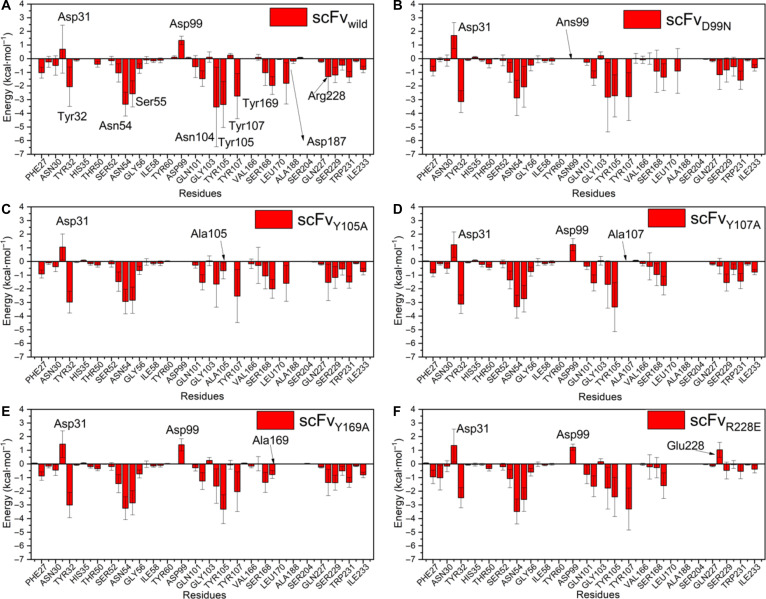
Per-residue binding free energy (Δ*G*_res) distribution of single-chain variable fragment (scFv) residues in interaction with CD20 across different systems. Key paratope residues with significant contributions are indicated: Tyr32 (CDR-H1), Asn54 and Ser55 (CDR-H2), Tyr102, Asn104, Tyr105, and Tyr107 (CDR-H3); in the VL domain, Tyr169 (CDR-L1), Asp187 (CDR-L2), Arg228, and Ser229 (CDR-L3). (A) scFvwild/CD20, (B) scFvD99N/CD20, (C) scFvY105A/CD20, (D) scFvY107A/CD20, (E) scFvY169A/CD20, and (F) scFvR228E/CD20. In (B) to (F), arrows indicate the positions of the mutated residues in each variant.

For a more detailed analysis, the per-residue effective binding free energy (Δ*G*_res) was calculated as proposed by Valdés-Tresanco *et al.* [66] allowing the identification of residues that contribute most to the scFv/CD20 interaction. The Δ*G*_res distributions (Fig. 1A to F) showed that, in all systems, the scFv residues involved in binding to CD20 at the interface were consistently observed across systems, with variations in their energetic contributions, while only a few residues displayed Δ*G*_res > 0. Notably, Asp31 exhibited Δ*G*_res > 0 in all systems, while Asp99 did so in 4 (Fig. 1A to F). Furthermore, the R228E mutation significantly altered Δ*G*_res, resulting in positive values (compare Fig. 1A to F). The tyrosine-to-alanine mutations led to an increase in Δ*G*_res, with Y107A reducing Δ*G*_res to zero, whereas Y105A showed a substantial increase, greater than that observed for Y169A (Fig. 1C to E).

The Δ*G*_res distribution analysis revealed that residues in the VH domain contributed more significantly to binding than those in the VL domain, particularly those located in CDR regions, such as Tyr32 (CDR-H1), Asn54 and Ser55 (CDR-H2), and Tyr102, Asn104, Tyr105, and Tyr107 (CDR-H3). In the VL domain, some residues also showed relevant contributions, including Tyr169 (CDR-L1), Asp187 (CDR-L2), Arg228, and Ser229 (CDR-L3).

**Fig. 2.**
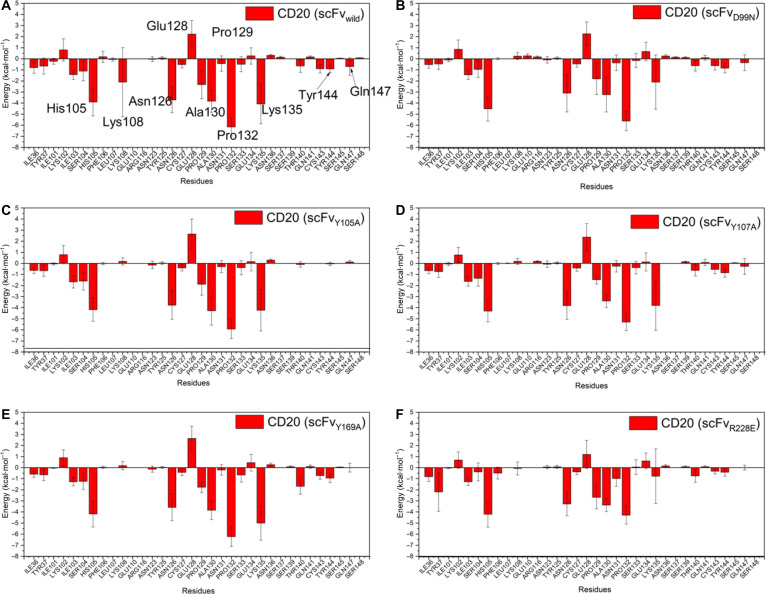
Per-residue binding free energy (Δ*G*_res) distribution of CD20 residues in interaction with the single-chain variable fragments (scFvs). The key epitope residues with significant contributions include His105, Lys108, Asn126, Glu128, Pro129, Ala130, Pro132, Lys135, Tyr144, and Gln147. (A) scFv_wild_/CD20, (B) scFvD99N/CD20, (C) scFvY105A/CD20, (D) scFvY107A/CD20, (E) scFvY169A/CD20, and (F) scFvR228E/CD20.

Figure 2A to F shows the Δ*G*_res distribution of CD20 residues, indicating their contribution to binding at the scFv/CD20 interface and highlighting residues that may constitute the epitope. The Δ*G*_res profiles revealed that the key CD20 residues involved in binding, for both scFv_wild_ and variant systems, were His105, Lys108, Asn126, Glu128, Pro129, Ala130, Pro132, Lys135, Tyr144, and Gln147 (MD numbering; −40 shift relative to cryo-EM and crystallographic structures, Fig. S3). All these residues exhibited negative Δ*G*_res values, except for Glu128. Among them, Lys108 and Lys135 were particularly noteworthy, with Lys135 showing a marked increase in Δ*G*_res in the D99N and R228E systems, mutations that involve charge alterations in the scFvs.

Figures S11A to F and S12A to F show the difference between the Δ*G*_res of each variant system and the reference scFv_wild_/CD20 system (ΔΔ*G*_res), for both the scFv (Fig. S11A to F) and CD20 (Fig. S12A to F). In Fig. S11B to F, positive ΔΔ*G*_res values indicate an increase in the residue’s binding free energy compared to the wild type (less favorable binding), whereas negative values (Δ*G*_res of the variant < Δ*G*_res of scFv_wild_) indicate a decrease, corresponding to more favorable binding of that residue. Values close to zero suggest that the mutation did not significantly affect the residue’s contribution to binding. For most residues, no relevant changes were observed (ΔΔ*G*_res ≈ 0); however, for those with greater variation, Δ*G*_res increased substantially, making the residue’s contribution to binding less favorable at the scFv/CD20 interface. The same procedure was applied to CD20, enabling the identification of critical binding residues at the interface.

**Fig. 3.**
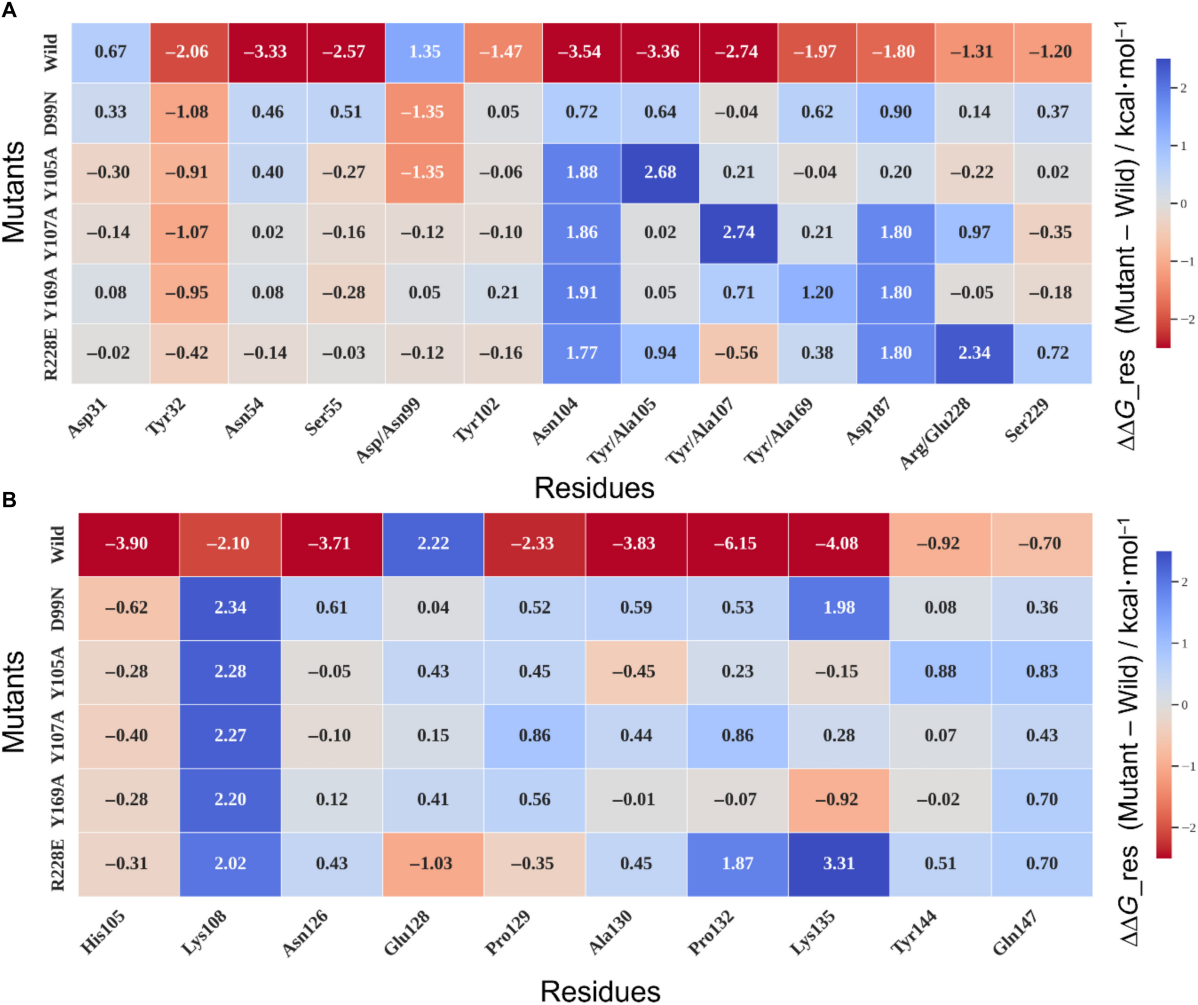
Heatmap representation of per-residue binding free energy changes (ΔΔ*G*_res) in single-chain variable fragment (scFv)/CD20 complexes. Δ*G*_res absolute values indicate whether a residue contributes favorably (negative) or unfavorably (positive) to binding (first row of the heatmap). ΔΔ*G*_res values were obtained by calculating the difference between the Δ*G*_res of each variant and that of the wild type (Δ*G*_res variant − Δ*G*_res wild) and were represented in heatmaps. The wild-type row was retained as a reference with absolute Δ*G*_res values. The scale ranges from −3.5 to +3.5 kcal·mol^−1^ (negative values in red and positive values in blue). The notation Asp/Asn99 indicates the substitution of Asp by Asn in the D99N mutation; the same applies to Y105A, Y107A, Y169A, and R228E. In (A), the columns correspond to scFv residues, showing correlations between scFv mutations and scFv residue contributions to CD20 binding. In (B), ΔΔ*G*_res values are calculated for CD20 residues, reflecting how scFv mutations impact the CD20 epitope.

**Fig. 4.**
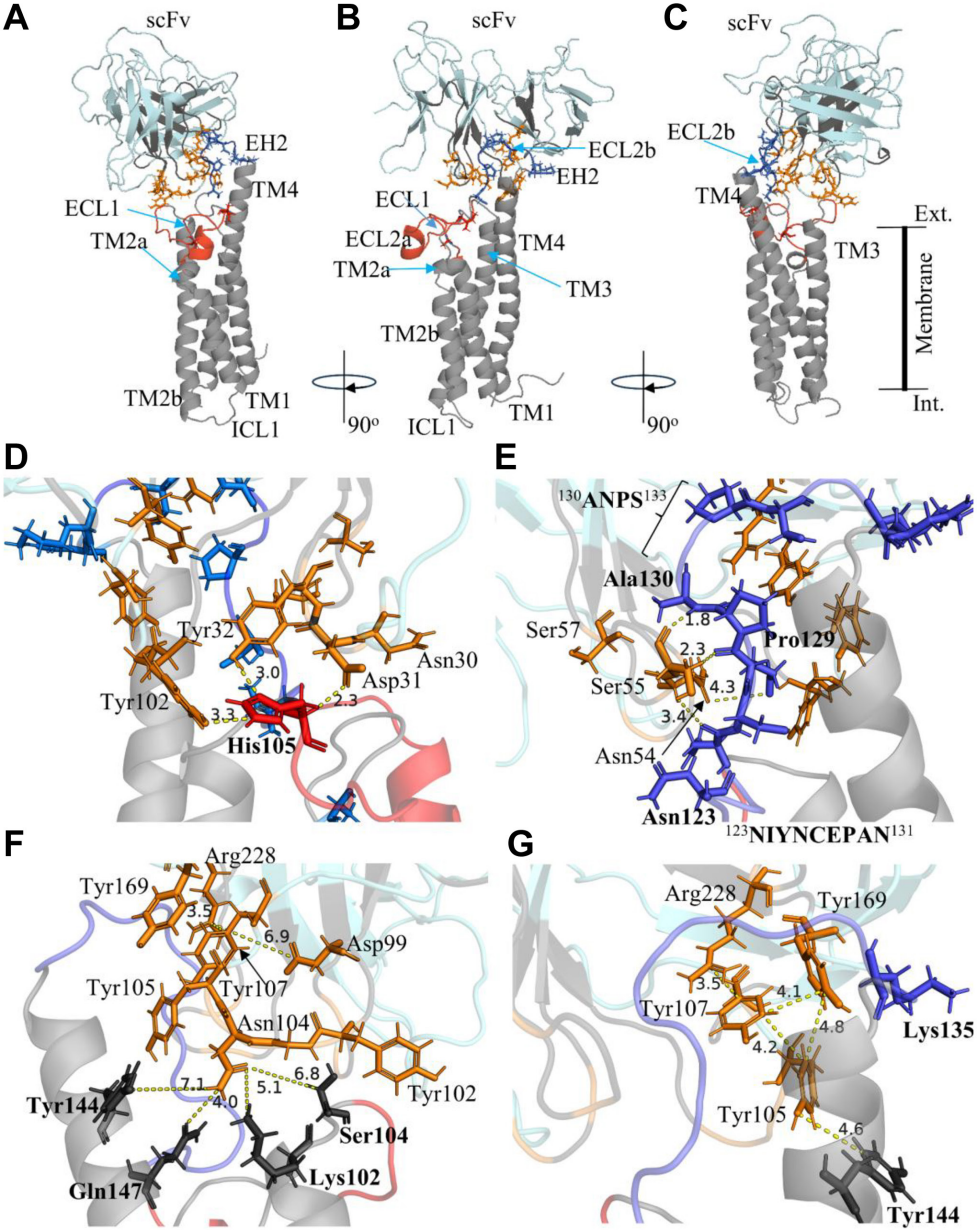
Structural organization of the CD20–ofatumumab single-chain variable fragment (scFv) complex and main interaction interfaces identified in molecular dynamics (MD) simulations. Structural representations shown were generated using the final configuration obtained from the MD trajectory of the scFv_wild_/CD20 system (700 ns). (A to C) Three rotated views (90° increments) of the CD20–scFv_wild_ complex showing the complete topology of monomeric CD20 (gray) and the orientation of the scFv (light blue). CD20 comprises 4 transmembrane helices (TM1 to TM4) and 2 extracellular loops (ECL1 and ECL2). ECL2 contains the antibody-binding epitope, subdivided into regions ECL2a (105 to 121) in red and ECL2b (122 to 131) in blue, flanked by extracellular helices EH1 and EH2 (^130^ANPS^133^), but in MD, EH1 and EH2 (134 to 136) do not appear to form helices. (D to G) Enlarged structural views highlighting specific paratope–epitope contacts. Protein structures are displayed in transparent cartoon representation. scFv residues are highlighted in orange and shown as sticks, while selected CD20 residues are shown as sticks. CD20 residues names are in bold; scFv residues in regular font. Distances between interacting residues are displayed in angstroms (Å) and indicated by yellow dashed lines. (D) Spatial distribution of ECL2 subregions showing ECL2b (blue) and ECL2a (red). The residue His105 of CD20 is highlighted within the ECL2a region. scFv residues Asn30, Asp31, Tyr32, and Tyr102 form contacts with the ECL2a region, particularly in proximity to His105, consistent with previously proposed structural models involving ECL2a interactions [35]. (E) View showing ECL2b positioned above the scFv surface, highlighting Pro129 and Ala130 of CD20. Pro129 is in proximity to Asn54 and Ser55, while Ala130 interacts with Ser57 and Ser55. The ANPS motif corresponding to the rituximab epitope region is indicated in the upper portion of the panel. (F) Membrane-proximal view highlighting Lys102 and Ser104 from TM3, together with Tyr144 and Gln147 from TM4, shown as black sticks. These 4 CD20 residues form contacts with Asn104 of the scFv and nearby phospholipid headgroups, as indicated by residue-level interaction analysis (Table S6). Tyr105 of the scFv is oriented toward the TM4 region and membrane-proximal surface. The measured Asp99–Arg228 distance (6.9 Å) indicates absence of salt-bridge formation at this interface. (G) Rotated view showing the relative positioning of TM4, bringing Lys135 into proximity with the beginning of the TM4 segment. In this orientation, ECL2b is positioned above the aromatic cluster formed by Arg228, Tyr169, Tyr107, and Tyr105. The relative position of Tyr105 with respect to Tyr144 indicates the approximate membrane level (not explicitly represented). Distances between residues support a cation–π interaction between Arg228 and Tyr107, with Tyr169 positioned in close proximity to this pair. The functional core of the ofatumumab epitope is consistent with previous structural models from Du *et al.* and Kumar *et al.* [35,39]. Note: Residue numbering corresponds to the truncated CD20 construct used in MD simulations; add +40 to match experimental numbering (Fig. S3).

Figure 3A and B shows heatmaps of the highlighted residues in the scFv and CD20, allowing correlation of variations in Δ*G*_res values of the scFv_wild_/CD20 system as a function of scFv mutations. The first horizontal row of the map shows the absolute Δ*G*_res values of the scFv_wild_/CD20 system, while the subsequent rows represent the difference in Δ*G*_res (ΔΔ*G*_res) for each variant relative to the reference system (scFv_wild_/CD20). Asp31 (CDR-H1) displayed a positive Δ*G*_res (~0.6 kcal·mol^−1^ in the wild type), confirming its low contribution to binding, a condition that remained unchanged in the variant systems. According to Table S6, Asp31 establishes occasional contacts with phospholipids (PLs) and with residues Lys108 and Arg116(CD20) in scFv_wild_/CD20 system; in the variants, this residue also forms a hydrogen bond (HB) with Ser104(CD20), located at the beginning of the large extracellular loop, near the membrane surface (ECL2a) (Fig. 4A to C).

Tyr32 (CDR-H1), which exhibited a negative Δ*G*_res in the scFv_wild_/CD20 system (−2.06 kcal·mol^−1^), consistently lost its contribution in all variants, failing to form contacts with CD20. As shown in Table S6, Tyr32 remains close to His105 in the ECL2a of CD20 but does not establish stable contacts (Fig. 4D).

Asn54 and Ser55 (CDR-H2) maintained stable and strongly negative values (−3.3 and −2.6 kcal·mol^−1^ in the wild type), reinforcing their high binding stability with CD20. According to Table S6, these residues interact with the 123–131 ECL2b region of the large loop (^123^NIYNCEPAN^131^) (Fig. 4E), with a high frequency of HBs observed between Asn126, Glu128, and Ala130 and Ser55 (CD20).

Asp99, located at the beginning of CDR-H3, showed low binding potential in the wild type (Δ*G*_res = +1.35 kcal·mol^−1^). In the scFv_Y105A_/CD20 and scFv_D99N_/CD20 systems, Δ*G*_res dropped to values close to zero (ΔΔ*G*_res = −1.35 kcal·mol^−1^), suggesting a functional correlation with Tyr105, as both belong to the same CDR-H3. In Table S6, Asp99 does not appear as a direct contact residue with CD20, reinforcing that its contribution is indirect and possibly modulated by proximity to Tyr105 within the CDR-H3 cluster.

Tyr102 has a Δ*G*_res of −1.42 kcal·mol^−1^, and its binding energy remains nearly unchanged across variants. It is very close to the membrane and interacts with PLs (Table S6). Asn104 has a highly negative binding energy (Δ*G*_res = −3.54 kcal·mol^−1^) and showed minor Δ*G*_res changes in scFv_D99N_/CD20 but significant increases in other variants. Asn104 may form contacts with residues from 2 different CD20 helices, including Lys102 and Ser104 (end of TM3 and beginning of the ECL2a large loop), as well as Tyr144 and Gln147 (TM4) (Fig. 4F), all close to the membrane surface. This residue is sensitive to spatially adjacent mutations such as Y105A, Y107A, Y169A, and R228E.

The residues Tyr105, Tyr107, and Tyr169 form a cluster that stabilizes the scFv_wild_/CD20 interface. When mutated, these residues showed increases in Δ*G*_res, corresponding to ΔΔ*G*_res values of 2.68, 2.74, and 1.20 kcal·mol^−1^, respectively, highlighting their essential role in CD20 binding (Fig. 4G) Tyr105 is membrane-proximal and forms HBs with Gln147(TM4) and interacts with Glu128 and Lys135(ECL2b), and Gln141(TM4). Tyr107 may form HBs with Glu128 and attractive interactions with Asn131(CD20) and Lys135(CD20). Tyr169 interacts with Lys135(CD20) in the variants and with Glu134(CD20) and Asn131(CD20) in the scFv_wild_/CD20 system. Asp187 also stands out for its Δ*G*_res variation in the scFv_Y107A_/CD20, scFv_Y169A_/CD20, and scFv_R228E_/CD20 systems, affecting the salt-bridge formation of Asp187-Lys135(CD20), observed in both the wild-type and variant complexes. It also interacts with membrane PLs. Arg228, with Δ*G*_res < 0, retained this value in most variants, except in the scFv_Y107A_/CD20 system, where the proximity to residue 107 and its mutation to Ala affected the interaction of Arg228 with CD20. The R228E mutation demonstrated that Arg228 plays an important role in stabilizing Tyr107, which belongs to the tyrosine cluster. Arg228’s side chain contacts the carbonyl group of Asn131(CD20) and the hydroxyl of Ser133(CD20), which are residues that form part of the rituximab epitope (^130^ANPS^133^). Ser229 did not directly influence cluster binding, exerting only a minor effect on Arg228 orientation. Contact analysis shows that Ser229 can form HBs with Glu134(CD20) and attractive interactions that are not observed in the Y169A and R228E variants.

Δ*G*_res correlations for CD20 residues as a function of scFv mutations were also analyzed (Fig. 3B and Table S7). His105, located at the beginning of the large loop (ECL2a) (Fig. 4D), had a Δ*G*_res of −3.90 kcal·mol^−1^ in scFv_wild_/CD20 and retained this value across variants. It is close to PLs and contacts Gln101 and Tyr102 (CDR-H3) (Table S7). Lys108, oriented toward the protein interior (Δ*G*_res = −2.10 kcal·mol^−1^), may stabilize the region via interactions with Asp30 and Asp31 (CDR-H1). In all variants, Lys108 showed reduced binding energy (ΔΔ*G*_res ~ 2 kcal·mol^−1^) and decreased attractive interactions with Asp30 and Asp31. Asn126 (Δ*G*_res = −3.71 kcal·mol^−1^ in scFv_wild_/CD20) exhibited unchanged ΔΔ*G*_res across variants, suggesting stable interaction with CDR-H2 (Trp53, Asn54, and Ser55), including HBs between side chains and backbones (scFv) (Fig. 4E).

Glu128 (Δ*G*_res = +2.22 kcal·mol^−1^) is in a region surrounded by hydrophobic residues and adjacent to the tyrosine cluster, and it can form HBs with the hydroxyl group of Tyr107 (CDR-H3), in addition to interacting with Trp53, Asn54 (CDR-H2) and Tyr105 (CDR-H3). In the R228E variant system, which involves a charge-reversal substitution, Glu128 showed a reduction in Δ*G*_res (ΔΔ*G*_res < 0), suggesting an improvement in the interface likely associated with local cluster destabilization. In contrast, the Y105A, Y107A, and Y169A mutations did not produce significant changes in Glu128’s ΔΔ*G*_res, indicating that the hydrophobic environment surrounding this residue remained largely unaltered.

Pro129 and Ala130 did not exhibit relevant ΔΔ*G*_res changes compared to the wild-type complex. These residues, together with Asn126 and Glu128, constitute the interaction region with CDR-H2 of the scFv. Pro132 (Δ*G*_res = −6.15 kcal·mol^−1^) is located above the CDR-L3 loop (where Arg228 is positioned). In the scFv_R228E_/CD20 system, mutation altered the loop conformation and slightly increased Δ*G*_res, thereby impairing binding. Ala130 is a key contact site with CDR-H2, forming HBs with Ser55 via main-chain carbonyl and amine groups (Table S7).

Lys135 showed an increase in Δ*G*_res in the scFv_R228E_/CD20 and scFv_D99N_/CD20 systems. This residue is located near the membrane (Fig. 4G), establishing contacts with PLs, as observed in most systems (Table S7). Table S7 also indicates that the range of contacts of Lys135 in the scFv_wild_/CD20 system was expanded in the variants, particularly in D99N and R228E, with additional interactions involving Tyr105, Tyr107, and Tyr169, which form the tyrosine cluster in the scFv. This closer proximity of Lys135 suggests that its interactions with the hydroxyl groups of these residues may contribute to destabilizing the cluster. Finally, Tyr144 and Gln147 were found to be minimally relevant for binding, with weak Δ*G*_res values. Tyr144 faces the membrane and interacts with PLs, while Gln147 forms HBs with Tyr105 (CDR-H3) (Fig. 4F).

Overall, the MD simulations indicate that ofatumumab scFv recognition is predominantly governed by interactions centered on the ECL2b region, particularly involving CD20 residues such as Asn126, Glu128, Pro129, Ala130, and Lys135, which interact with key paratope residues including Tyr105, Tyr107, Tyr169, and Arg228. In contrast, residues from the ECL2a region, such as His105 and Lys108, displayed secondary contributions, while ECL1 showed only limited and transient contacts. These structural observations provided the rationale for the subsequent experimental validation through site-directed mutagenesis and flow cytometry assays.

### *In vitro* results

#### Expression and purification of scFv_wild_ and variants in *E. coli* Shuffle B T7

After site-directed mutagenesis, single colonies were picked and analyzed by Sanger DNA sequencing to confirm the presence of expected mutations. The transformation of *E. coli* Shuffle B strains was successfully performed via heat shock, aiming to the introduction of pET-SUMO plasmids harboring the genes of interest, corresponding to scFv_wild_ and its variants. The efficiency of the process was confirmed by the emergence of positive colonies, which were cultured in LB medium followed by induction of recombinant protein expression.

Following the expression and purification steps, the presence of scFv_wild_ and its variants was confirmed, as shown in Figs. S13 to S18. The construct containing the SUMO tag exhibited an apparent molecular weight of approximately 41 kDa. Upon SUMO tag removal, the isolated scFv_wild_ displayed a molecular weight of 26 kDa, as verified by the SDS-PAGE shown in Fig. S14D.

The scFvs were produced in a total volume of 250 ml of culture. At the end of the purification process, a final volume of 2 ml of purified protein was obtained, with a concentration determined at 0.200 mg/ml, totaling 0.4 mg of protein. Based on this result, the total yield of expression and purification was 1.6 mg/l of culture. This value reflects the efficiency of the adopted production system and serves as a comparative parameter for future optimizations of the protocol, aiming at increasing productivity and recovery of the recombinant protein. Ofatumumab mutants had variable yields, although all superior to the 10 μg/ml concentration used for the flow cytometry experiments.

#### Flow cytometry analysis of scFv_wild_ and variants binding to CD20 on the cell surface

Flow cytometry analysis showed that the scFvs derived from ofatumumab could interact with the CD20 protein expressed on the cell surface. The gating strategy used to identify and select the population of interest is shown in Fig. S17. The binding of all scFvs to Raji cells was measured using 1,000 ng in a total volume of 100 μl to allow comparison between wild type and mutants. Flow cytometry measurements were performed at a single concentration, thus rMFI values should be interpreted as qualitative indicators of residue functional importance rather than quantitative affinity measurements. A commercial phycoerythrin-conjugated anti-CD20 mAb (BD Pharmingen, clone 2H7, cat. 555623) was used as a positive control, confirming the expression of the CD20 antigen on Raji cells. Jurkat cells, lacking CD20 expression, served as a negative control and showed no detectable fluorescence with the scFvs (Fig. S18).

**Fig. 5.**
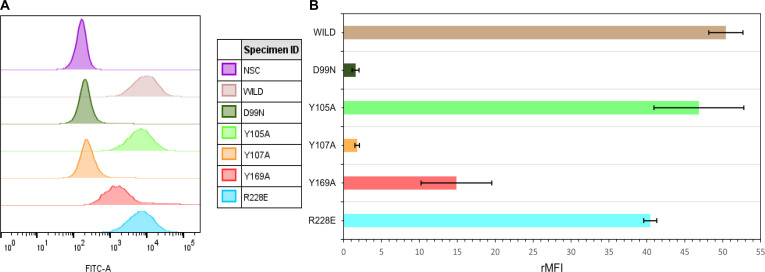
Flow cytometry analysis of Raji cells (CD20^+^) incubated with 1,000 ng of ofatumumab single-chain variable fragment (scFv) wild type and variants. (A) Histogram plots of CD20 staining by scFvs. (B) Relative mean fluorescence intensity (rMFI) of cells incubated with scFv type (type = WILD, D99N, Y107A, Y169A, and R228E). MFI values were measured and normalized to the NSC to generate rMFI values. Data represent mean ± SD from 2 independent experiments performed in triplicate. WILD, wild ofatumumab scFv; NSC, nonspecific control.

The histograms generated in the experiments represent the distribution of fluorescence intensity across the cell population as a function of specific binding of the fluorescent marker (Fig. 5A). The observed fluorescence, mediated by the FITC fluorophore, directly reflects the interaction between the scFvs and Raji cells. Consistent with their lack of CD20 expression, Jurkat cells exhibited no fluorescence when incubated with the scFvs.

Among the analytical parameters used in flow cytometry, the rMFI is particularly noteworthy (Fig. 5B). MFI can be calculated as the arithmetic mean, geometric mean, or median of the fluorescence signal, reflecting the average amount of fluorescent marker per cell within a given sample. rMFI represents the normalization of the sample MFI in relation to the control, enabling direct comparison across different experimental conditions. This approach is particularly valuable for minimizing technical variability between independent assays and for accurately detecting changes in the expression or abundance of the target molecule [68]. Formally, rMFI is defined as the ratio of the MFI of the sample of interest to the MFI of the corresponding control. In the present study, rMFI was employed to compare the binding of scFv_wild_ and its point variants to CD20, allowing evaluation of how each mutation affects antigen recognition while accounting for interassay variability.

In the conducted analyses, Jurkat cells consistently exhibited low rMFI values, consistent with the absence of specific labeling. In contrast, Raji cells incubated with scFv_wild_ showed a significant increase in fluorescence intensity, indicating strong interaction with CD20. This pattern was even more pronounced in the rMFI analysis.

Raji cells treated with variant scFv_Y105A_ (46.9 ± 5.9) and scFv_R228E_ (40.4 ± 0.9) resulted in a slight decrease in scFv binding to CD20 compared with the wild-type scFv (50.4 ± 2.3). In contrast, the Y107A (1.8 ± 0.3), D99N (1.6 ± 0.5), and Y169A (14.9 ± 4.7) mutations showed a marked reduction in rMFI relative to the wild-type scFv. These results suggest that the introduced mutations impair CD20 binding, underscoring the critical role of these residues in antigen recognition. Among all tested variants, scFv_Y107A_ and scFv_D99N_ compromised CD20+ cell binding, as shown in Fig. 5, suggesting that the residues identified as critical for the interaction of ofatumumab scFv with CD20 in the in silico analysis are also confirmed in the *in vitro* tests.

## Discussion

The present study provides a structural and functional characterization of the ofatumumab–CD20 interaction by integrating MD simulations, mutational analysis, and flow cytometry measurements. Unless otherwise stated, the residue numbering follows the MD model, which is shifted by −40 residues relative to the cryo-EM structure of CD20 [38]. Our results provide structural evidence consistent with a membrane-proximal epitope centered on ECL2b and EH2, supporting the model proposed by Kumar *et al.* In this context, earlier models derived from binding assays and structural analyses of the ofatumumab Fab suggested alternative epitope configurations involving the extracellular loops of CD20, which have been interpreted differently across studies [7,35–37]. This membrane-proximal epitope positioning was associated with the high binding affinity and strong complement-dependent cytotoxicity activity reported for ofatumumab in these early functional studies.

These early interpretations originated from functional and mutagenesis studies conducted prior to the availability of membrane-resolved CD20 structures. Initial work by Teeling *et al.* [37] demonstrated, through peptide competition assays, that antibody binding could be inhibited by extracellular loop–derived peptides, supporting the proposal of a dual-loop epitope involving ECL1 and ECL2a. Subsequent mutagenesis studies further reinforced the involvement of residues located N-terminal to the ANPS motif (Teeling *et al.* [7]). In parallel, Du *et al.* [35] reported the crystal structure of the ofatumumab Fab and attempted cocrystallization with peptides corresponding to ECL1, ECL2a, and ECL2b but obtained crystals of the Fab alone, suggesting that antigen recognition depends on a conformational epitope requiring the structural integrity of CD20 extracellular domains.

This historical framework was later refined when the cryo-EM study by Kumar *et al.* [38] reevaluated the CD20–ofatumumab complex using full-length CD20 reconstituted in lipid bicelles, thereby preserving the membrane environment and revealing additional structural features: a dimeric organization of CD20 (subunits A and B), the presence of 4 transmembrane helices (TM1 to TM4), and distinct orientations of the extracellular loops (ECL1 and ECL2) compared to the model of Du *et al.* [35]. Under this more physiologically relevant context, Kumar *et al.* [38] proposed that the ofatumumab epitope is centered on ECL2b (residues 122 to 131), near the ANPS motif, and is therefore more like that of rituximab, contrasting with the earlier dual-loop (ECL1 + ECL2a) model proposed by Teeling *et al.* [7,37]. Given the structural variability among previously proposed models, we conducted MD simulations of the scFv_wild_/CD20 complex and scFv variants targeting key residues. Complementary simulations of scFv_wild_ and its variants in aqueous solution were also performed to clarify the molecular basis of binding and evaluate intrinsic conformational rearrangements among CDRs that may influence residue-level stability and interface formation. Such structural rearrangements have been previously shown to affect antigen recognition in scFv systems [69].

In the MD simulations of native ofatumumab, the main interaction region was located within ECL2b, whereas contact with ECL1 occurred only through the transient lateral approach of Asn30 and Trp53 side chains from the scFv to Ile36 and Tyr37 of CD20, as shown in Fig. S19B. The minimum-distance plots between these residue pairs (Fig. S19A) showed values below 0.25 nm, consistent with close spatial proximity. However, these interactions were predominantly hydrophobic and transient, indicating a secondary steric contribution rather than a primary role in antigen recognition. The hydroxyl group of Tyr37 may interact attractively with the indole nitrogen of Trp, an interaction not captured by the HB metric used to define contacts in Tables S6 and S7. Nevertheless, the Δ*G*_res values indicate that these residues contribute energetically to binding, as shown in Figs. 1A and 2A.

The MD observations preserve the initial contacts described by cryo-EM in the work of Kumar *et al.* [38], which demonstrated that ofatumumab interacts with ECL2b residues Tyr121, Asn126, and Glu128, as well as Ala130 and Asn131 located at the tip of EH2, near TM4. These interactions define the functional core of the ofatumumab epitope, restricted to a single CD20 subunit, but with expanded contact regions in the MD model, suggesting greater flexibility and a broader coupling interface between the scFv and the CD20 surface.

The study by Teeling *et al.* [37] demonstrated, through peptide competition and immunization assays, that antipeptide antibodies corresponding to ECL1 blocked the binding of the human antibodies 2F2 and 7D8, which later gave rise to ofatumumab. This experimental evidence is consistent with the MD results, in which Ile36 and Tyr37 of CD20 form lateral contacts with the ofatumumab scFv, suggesting that occupation of ECL1 by another antibody would sterically hinder ofatumumab binding.

In a subsequent work, Teeling *et al.* [7] confirmed by mutagenesis that the epitope of human anti-CD20 antibodies lies N-terminal to the ANPS motif, corresponding to the ECL2a region, and includes the small extracellular loop ECL1. In that study, mutations within ECL2a markedly reduced antibody binding, but reactivity was restored when human ECL1 and ECL2a segments were inserted into murine CD20, supporting the dual-loop model later refined by Kumar *et al.* [38] into an ECL2b-centered epitope. These observations, supported by the dynamic behavior observed in MD simulations, further reinforce the central role of ECL2b as the primary interaction region, while suggesting that ECL1 contributes mainly through transient lateral contacts that influence spatial positioning rather than direct antigen recognition.

The study by Du *et al.* [35] presented the crystal structure of the ofatumumab Fab fragment in the absence of antigen. The authors reported that they attempted to cocrystallize the Fab with 3 peptides corresponding to ECL1, ECL2a, and ECL2b of CD20, but all attempts failed, yielding crystals of the Fab alone. They interpreted this as evidence that ofatumumab recognizes a conformational epitope requiring the structural integrity of both ECL1 and ECL2, consistent with the proposal of Teeling *et al.* [7].

In the same work, Du *et al.* [35] described that the 6 CDR loops of the ofatumumab Fab form a large hydrophobic pocket, which constitutes the putative antigen-binding site. The CDR-L3, CDR-H1, CDR-H2, and CDR-H3 loops predominate, highlighting hydrophobic residues TyrL32, TrpL94, TrpH53, IleH58, TyrH60, TyrH102, and TyrH105 (corresponding to Tyr169, Trp231, Trp53, Ile58, Tyr60, and Tyr105 to 107 in MD), which line the periphery of the pocket. A positively charged residue, ArgL91 (Arg228 in MD), is located at the pocket’s base. Within this structural framework, mutations introduced at selected interface residues enabled the evaluation of local perturbations relative to the scFv_wild_/CD20 system. Complementary MD simulations of scFv_wild_ and its variants in aqueous solution were performed to examine structural rearrangements within the CDR regions in the absence of antigen. Similar rearrangements have been shown to impact antigen recognition in scFv systems [69]. These simulations established a structural reference framework for the interpretation of subsequent experimental binding measurements.

The recombinant expression of the ofatumumab scFv and its variants was performed in a prokaryotic system using a SUMO fusion tag to enhance solubility and facilitate affinity purification. This strategy enabled the production of sufficient and highly pure quantities of the scFv (Fig. S16), allowing their use in functional assays to evaluate CD20 binding by flow cytometry.

Flow cytometry results revealed a binding profile consistent with the structural and energetic variations obtained from MD simulations, performed both at the scFv/CD20 interface and in aqueous solution, reinforcing the model of an epitope centered on ECL2b, supported by hydrophobic and electrostatic interactions within the ofatumumab paratope. The scFv_wild_ showed the highest fluorescence intensity (rMFI = 50.4 ± 2.3), consistent with its strong binding estimated by MMPBSA (Δ*G*_binding_ = −34.5 ± 15.6 kcal·mol^−1^). Although the average Δ*G*_binding_ for scFv_Y107A_/CD20 (−34.4 ± 10.9 kcal·mol^−1^) was similar to that of the scFv_wild_/CD20 complex, this variant exhibited an almost complete loss of cellular binding (rMFI = 1.80 ± 0.3), indicating that the effect of the mutation is primarily structural rather than thermodynamic and reinforcing the notion that antibody–antigen interactions are highly sensitive to local residue composition at the binding interface [70]. This apparent discrepancy reflects the fact that Δ*G*_binding_ evaluates the stability of the preformed complex, rather than the efficiency of its initial association. The remaining variants (Y105A, R228E, and Y169A) displayed progressively reduced binding capacity (rMFI = 46.9, 40.4, and 14.9, respectively). The scFv_D99N_/CD20, with Δ*G*_binding_ = −13.4 ± 14.5 kcal·mol^−1^, also showed minimal binding to scFv_D99N_ variant (rMFI = 1.6 ± 0.5).

Notably, the shorter Arg228–Asp99 distance observed for the Y107A variant (0.30 ± 0.17 nm), compared to Y169A (0.42 ± 0.25 nm), suggests a higher propensity for salt-bridge formation in Y107A in aqueous solution (Fig. S7J and K). The formation of this interaction promotes increased torsional displacement of CDR-H3 (Fig. S7D and E), indicating that enhanced electrostatic coupling between Arg228 and Asp99 can restrict the intrinsic flexibility of this loop (Table S4). In the wild-type structure, stabilization of CDR-H3 is supported by an HB between the carboxyl group of Asp99 and the backbone amine of Tyr107, as observed in the interface simulations (Fig. S19C to F), where this interaction maintains the native orientation of Tyr107 within the aromatic cluster. The consistency between the stabilizing interactions observed in the interface environment and the conformational tendencies detected in aqueous simulations supports a cooperative structural mechanism governing CDR-H3 organization across both contexts.

In addition to this hydrogen-bond stabilization, Arg228 plays a complementary structural role through π–cation interactions with aromatic residues Tyr107 and Tyr169, as indicated by the measured inter-residue distances reported in Fig. S7. These interactions contribute to maintaining the spatial organization of the aromatic cluster spanning CDR-H3 and neighboring light-chain loops, reinforcing structural coupling between VH and VL domains (Fig. S7 A to F).

In contrast, enhanced Arg228–Asp99 proximity in scFv_Y107A_ and scFvY_169A_ appears to reduce the natural flexibility of CDR-H3 (Fig. S7 D and E and Table S4), favoring torsional displacement of the CDRs and generating conformational states less compatible with productive interface formation. These observations are consistent with residue-level energy decomposition analyses in scFv/CD20 system, indicating that Asp99 contributes indirectly to antigen recognition by stabilizing Tyr107 positioning within the CDR-H3 cluster, while Arg228 supports the structural integrity of the aromatic network through π-mediated interactions.

Energy decomposition (Δ*G*_res) indicates that the main role of Asp99 is to stabilize the positioning of Tyr107 through an HB between the carboxyl oxygen of Asp99 and the backbone amine of Tyr107 (Fig. S19C to F). This interaction stabilizes the orientation of CDR-H3, ensuring the correct positioning of Tyr107 within the aromatic cluster. General interface residues involved in stabilizing interactions are frequently observed to form energetic hotspots in antibody–antigen complexes [34]. Additionally, the proximity of opposite charges between Asp99 and Arg228 promotes occasional salt-bridge formation (Fig. S19D), contributing to rigidity and coupling between the light and heavy chains. Similar cooperative structural effects influencing CDR positioning and antigen recognition have been reported in scFv systems following subtle structural modifications [69]. Because the mutations were introduced directly into preformed scFv/CD20 complexes, the MD simulations reflect the local and cooperative effects of substitutions without including the full association and conformational rearrangement process. Therefore, Δ*G*_binding_ values represent internal complex energies and do not correlate linearly with the experimental binding affinities observed by flow cytometry.

The observed functional hierarchy (Y107 > D99 > R228 > Y169 > Y105) reflects the structural contribution of each residue in stabilizing the tyrosine cluster (Y105–Y107–Y169) and the electrostatic anchor of Arg228. Structurally, the MD and flow cytometry findings align closely with the cryo-EM model of Kumar *et al.* [38], which positioned the ofatumumab epitope within the ECL2b loop (residues 121 to 131) and the tip of EH2, involving Tyr121, Asn126, Glu128, Ala130, and Asn131. However, the simulations reveal that the contact region extends from Ala130 to Tyr144, reaching the start of TM4, where the scFv tyrosine cluster (Y105–Y107–Y169) interacts beneath ECL2b, while the CDR-L3 lies above the ^130^ANPS^133^ region, corresponding to the rituximab epitope (Fig. S19). This overlap suggests that, although ofatumumab and rituximab partially share the same CD20 surface, ofatumumab adopts a distinct orientation, with CDR-L3 anchored over the ANPS motif and the aromatic cluster stabilizing the ECL2b/EH2 interface.

The mutations Y105A, Y107A, and Y169A, associated with the tyrosine cluster, reduced binding capacity primarily through loss of hydrophobic packing and π–π interactions directed toward ECL2b and EH2. Together with lateral π–π stacking between tyrosines and π–cation interactions (Arg228–Tyr107), these observations confirm ECL2b/EH2 as the dominant functional core for recognition, in contrast to ECL1. This conclusion agrees with the observations of Kumar *et al.* [38] but contrasts with the dual-loop model (ECL1 + ECL2a) proposed by Du [35] and Teeling [7].

The studies by Teeling *et al.* [7,37] originally proposed the dual-loop model based on peptide competition and mutagenesis experiments, showing that anti-peptide antibodies targeting ECL1 blocked the binding of human antibodies that preceded ofatumumab. Although initially interpreted as direct involvement of ECL1, our results suggest that this blockage is more likely steric rather than due to direct interaction, since simulations reveal only transient, lateral contacts between Trp53/Asn30 (scFv) and Ile36/Tyr37 (CD20) (Fig. S19A and B). Thus, the data presented here remain compatible with Teeling’s findings but restrict the role of ECL1 to spatial orientation of the scFv.

Du *et al.* [35] reported the crystal structure of the ofatumumab Fab without bound antigen and noted unsuccessful cocrystallization attempts with peptides corresponding to ECL1, ECL2a, and ECL2b. The authors concluded that antigen recognition is conformational and dependent on the integrity of the extracellular loops, a conclusion consistent with our findings. The inability to bind isolated peptides reinforces the need for a cooperative conformation between ECL2b and EH2, the same region identified in our simulations and validated experimentally by the loss of binding in Y107A, Y169A, and R228E variants. The hydrophobic arrangement described by Du in the paratope, TyrL32, TrpL94, TrpH53, IleH58, TyrH60, TyrH102, TyrH105, and ArgL91, largely corresponds to the residues identified in our MD simulations (Tyr169, Trp231, Trp53, Ile58, Tyr60, Tyr105 to 107, and Arg228), indicating structural convergence between the crystallographic and MD-derived models.

Collectively, our structural, energetic, and functional analyses converge to define a refined model of the ofatumumab–CD20 interface. The simulations and mutational data demonstrate that the antibody recognizes a conformational epitope centered on ECL2b and extending toward the membrane-proximal EH2 helix, stabilized by the aromatic triad Y105–Y107–Y169 and the electrostatic anchor R228, consistent with the critical role of localized interaction hotspots in stabilizing antibody–antigen interfaces [70]. The light-chain CDR-L3 lies directly above the ANPS motif, a region also contacted by rituximab, but ofatumumab adopts a distinct orientation that engages both ECL2b and EH2 through hydrophobic and π–cation stacking interactions. This configuration may contribute to the higher complement activation efficiency reported for ofatumumab, likely due to its membrane-proximal epitope positioning and stable interface organization, as described in previous structural studies [37]. By integrating experimental and computational data, our study bridges the historical dual-loop model (ECL1 + ECL2a) proposed by Teeling and Du with the membrane-embedded architecture revealed by Kumar’s cryo-EM structure, contributing to a unified structural understanding of CD20 recognition by ofatumumab.

## Conclusion

In this work, we addressed the long-standing question of the precise molecular basis for ofatumumab recognition of CD20. Through atomistic MD simulations, free-energy decomposition, and site-directed mutagenesis validated by flow cytometry, we demonstrate that the functional epitope of ofatumumab is primarily centered at the ECL2b/EH2 interface, rather than spanning both extracellular loops as previously proposed. The interaction is governed by a cooperative network of hydrophobic and electrostatic contacts, dominated by the Y105–Y107–Y169 tyrosine cluster and R228, that stabilizes the paratope architecture and supports stable antigen recognition. These findings help reconcile previous structural discrepancies and provide a coherent framework for the rational exploration and optimization of next-generation anti-CD20 therapeutics, including scFv-based CAR-T constructs, and engineered antibody fragments optimized for enhanced stability, specificity, and effector function.

## Data Availability

All molecular structures, parameter files, and input and output files used in this study are publicly available in a GitHub repository at the following link: https://github.com/marcos-lourenzoni-gepess/CSBJ. These files allow full reproduction of the computational setup and analyses described in this work.
