# Supplementary material for: Revisiting the Ofatumumab Epitope on CD20 through Integrative Molecular Dynamics and Flow Cytometry Analyses
Source: Comput Struct Biotechnol J. 2026 May 25;35(1):0096. doi: 10.34133/csbj.0096 (PMC13199643; doi:10.34133/csbj.0096)
Supplement: Supplementary 1 — Figs. S1 to S19 Tables S1 to S7 [file csbj.0096.f1.docx]

**SUPPLEMENTARY DATA**

**Figure S1.** **Amino acid sequence of the scFv_wild_, with residue numbering and domain annotation.** The variable heavy (VH) domain is shown in green, the linker region in black, and the variable light (VL) domain in red. Mutated residues are highlighted in bold and underlined: D99, Y105, Y107, Y169, and R228. The CDRs are indicated as underlined regions in the sequence. Residues of CDR-H1: 30–35; CDR-H2: 50–66; CDR-H3: 99–111; CDR-L1: 161–171; CDR-L2: 187–193; CDR-L3: 226–234.


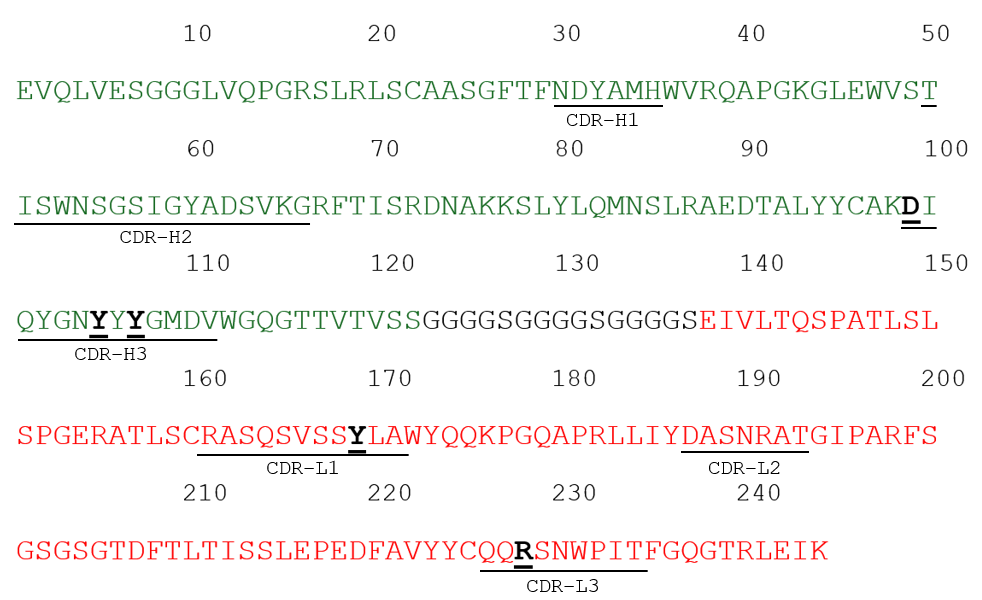


**Figure S2. Amino acid alignment of the wild-type and variant scFv sequences.** The variable heavy (VH) domain is shown in green, the linker region in brown, and the variable light (VL) domain in red. Mutation sites are indicated by arrows and highlighted in yellow, while the substituted residues are emphasized in black font (D99, Y105, Y107, Y169, and R228). Alignment was performed using Clustal Omega [1], and residue coloring was generated with ESPript 3.0 [2].


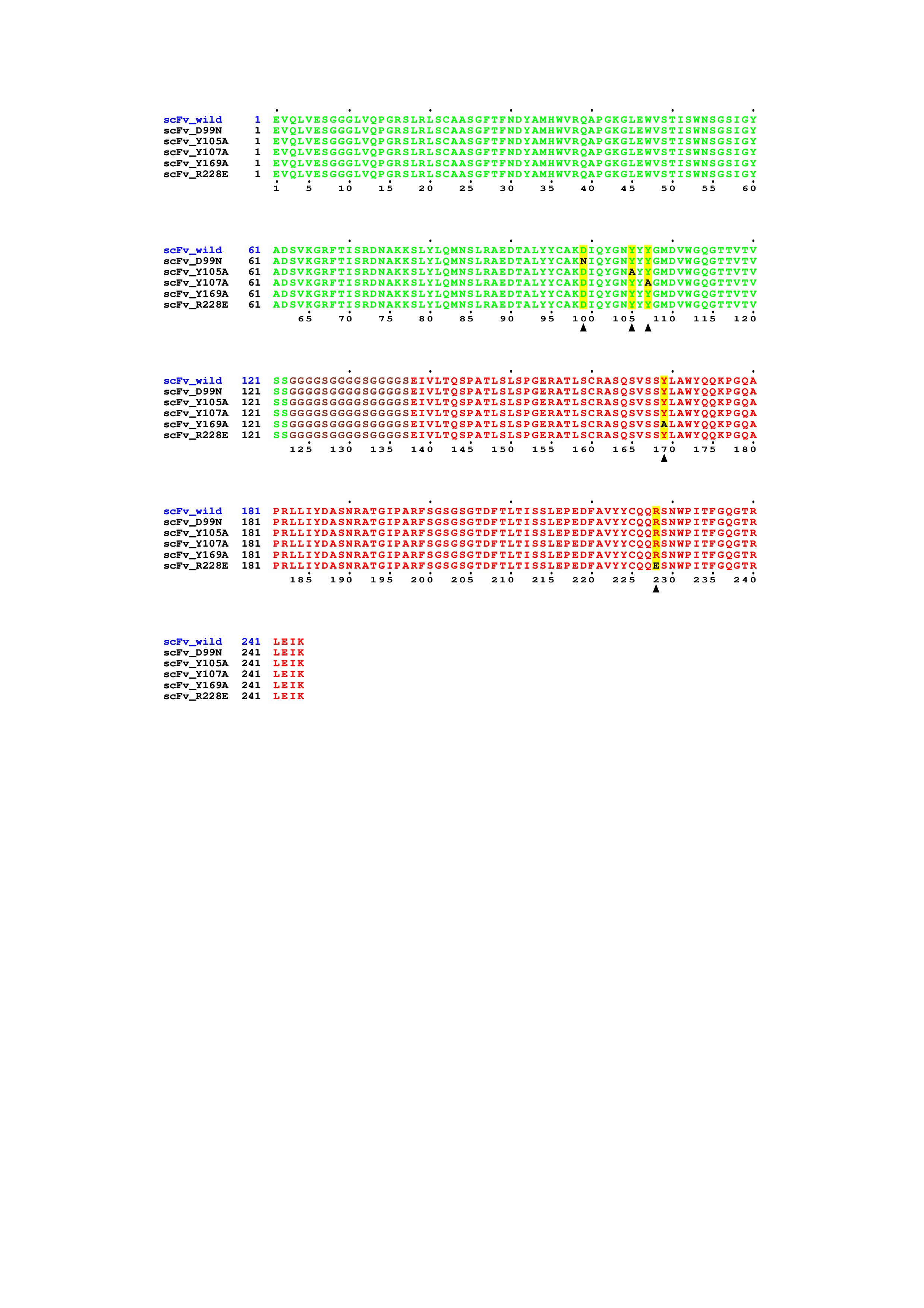


**Figure S3. Sequence alignment of the CD20 used in this study and the reference sequence obtained from UniProt.** Identical residues are highlighted with a red tag. The alignment was performed with Clustal Omega [1], and residue coloring was generated with ESPript 3.0 [2].


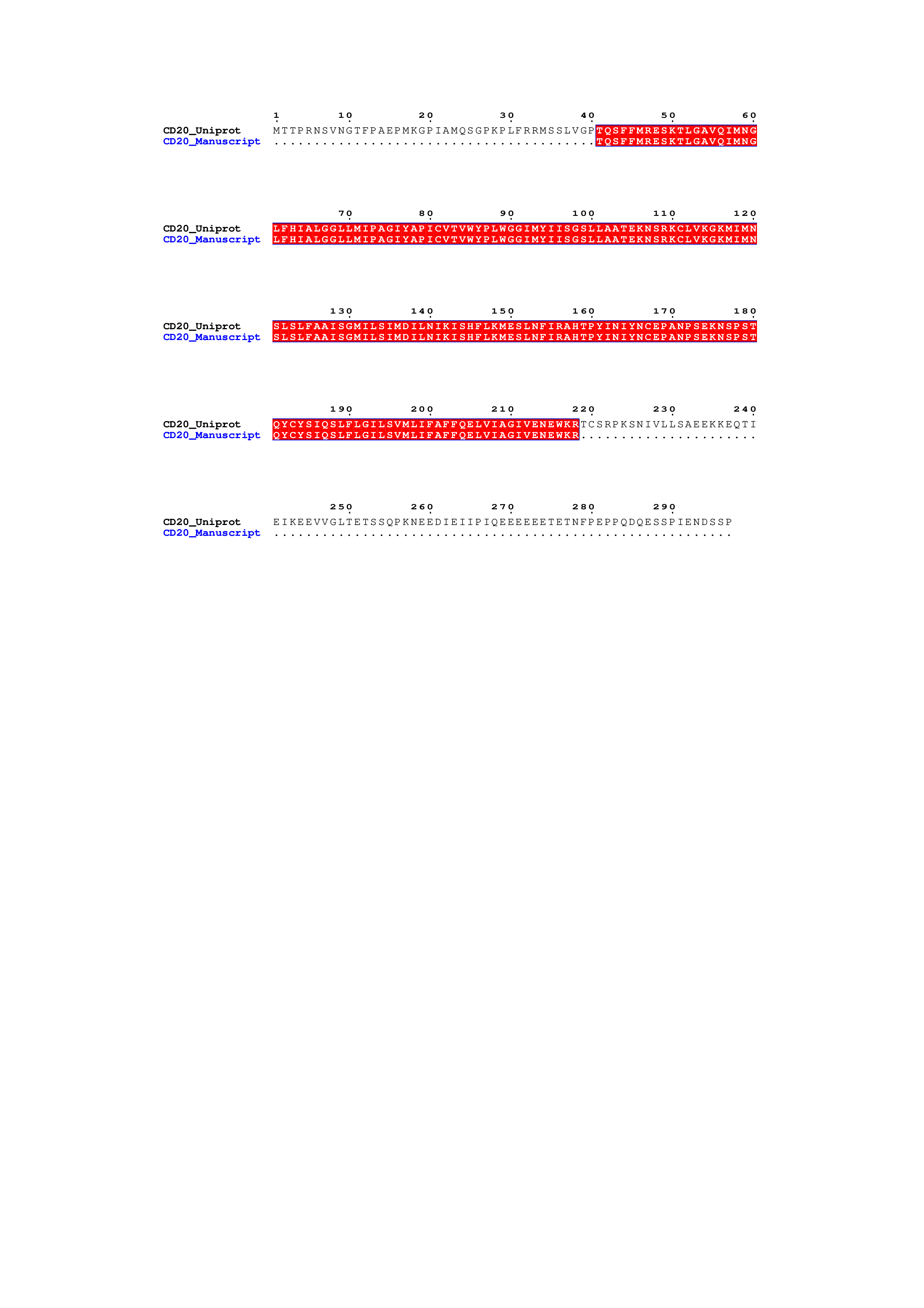


Note: The CD20 sequence used in this study lacks the N-terminal cytoplasmic segment, leading to a -40 residue shift relative to the canonical numbering. To match the numbering reported in Du (2009) and Kumar (2020)[3,4], add +40 residues to the positions shown in the alignment.

**Table S1. Simulated systems composition, detailing the system nomenclature, number of water molecules and ions, dimensions, and types of simulation boxes**. All the systems consist of the scFvs (wild-type or variants) simulated either in interaction with the CD20 immersed in a lipid bilayer in a box filled with water molecules or as isolated scFv structures in aqueous solution. Systems containing CD20 were designated as scFv/CD20, including the native complex (scFv_wild_/CD20) and the mutant variants scFv_D99N_/CD20, scFv_Y105A_/CD20, scFv_Y107A_/CD20, scFv_Y169A_/CD20 and scFv_R228E_/CD20. Corresponding isolated systems in aqueous solution were designated as scFv_wild_ and scFv_D99N_, scFv_Y105A_, scFv_Y107A_, scFvY169A, and scFv_R228E_

| **System**  **scFv/CD20** | **Number of water molecules** |  | **Na^+^** | **Cl^-^** | **Simulation box dimensions** nm (format) |
| --- | --- | --- | --- | --- | --- |
| scFv_wild_/CD20 | 65273 |  | 179 | 185 | 13x13x16 (rectangular) |
| scFv_D99N_/CD20 | 64824 |  | 177 | 184 | 13x13x16 (rectangular) |
| scFv_Y105A_/CD20 | 64777 |  | 178 | 184 | 13x13x16 (rectangular) |
| scFv_Y107A_/CD20 | 64825 |  | 178 | 184 | 13x13x16 (rectangular) |
| scFv_Y169A_/CD20 | 64817 |  | 178 | 184 | 13x13x16 (rectangular) |
| scFv_R228E_/CD20 | 64815 |  | 178 | 183 | 13x13x16 (rectangular) |
| scFv_wild_ | 20944 |  | 61 | 63 | 1.8 (dodecaedron) - distance between the protein edges and the box edges in all axes |
| scFv_D99N_ | 22413 |  | 65 | 68 | 1.8 (dodecaedron) - distance between the protein edges and the box edges in all axes |
| scFv_Y105A_ | 22417 |  | 65 | 67 | 1.8 (dodecaedron) - distance between the protein edges and the box edges in all axes |
| scFv_Y169A_ | 22414 |  | 65 | 67 | 1.8 (dodecaedron) - distance between the protein edges and the box edges in all axes |
| scFv_R228E_ | 22417 |  | 65 | 65 | 1.8 (dodecaedron) - distance between the protein edges and the box edges in all axes |

**Table S2. Description of the procedures used during the thermalization and production phases of the MD simulations, including the duration of each NVT and NPT step during thermalization, as well as the total trajectory acquisition time for each system.** The dataset comprises both membrane-embedded scFv/CD20 complexes and isolated scFvs simulated in explicit aqueous solvent. System with the native scFv: scFv_wild_/CD20. All the systems with the scFvs variants (scFv_D99N_/CD20, scFv_Y105A_/CD20, scFv_Y107A_/CD20, scFv_Y169A_/CD20 and scFv_R228E_/CD20) were referred in this table as ‘Variants/CD20’ since the protocol was the same for all of them. The systems were initially subjected to three thermalization steps under NPT conditions, followed by three steps under NVT, and then an additional four under NPT. Throughout all these stages, positional restraints were applied to both the protein and the membrane. Finally, the last thermalization step was performed under NPT conditions without restraints, to prepare the system for the subsequent trajectory production. Isolated scFvs are collectively referred to as ‘scFvs*’*, as the same simulation protocol was employed for all systems (scFv_wild_ and scFv_D99N_, scFv_Y105A_, scFv_Y107A_, scFv_Y169A_, and scFv_R228E_).

| **Systems** | **Thermalization time** (ns)  **(Ensemble NVT or NPT)** | **Trajectory acquisition time (**ns) |
| --- | --- | --- |
| scFv_wild_/CD20 | 1.125 with restriction (NPT) | 700 (NPT) |
|  | 0.375 with restriction (NVT) |  |
|  | 101.5 with restriction (NPT) |  |
|  | 0.5 without restriction (NPT) |  |
| Variants/CD20 | 1.125 with restriction (NPT) | 200 (NPT) |
|  | 0.375 with restriction (NVT) |  |
|  | 101.5 with restriction (NPT) |  |
|  | 0.5 without restriction (NPT) |  |
| scFvs | 0.0125 with protein frozen (NVT) | 300 (NPT) |
|  | 0.005 without restriction (NVT) |  |
|  | 0.1 without restriction (NPT) |  |

**Table S3**. **Nucleotide sequence of the primers used to insert mutations into the Ofatumumab scFv gene.**

| **scFv_Y107A_** | Fw:TACTATGCGGGCATGGACGTTTGGGGCCAAG  Rv:CATGCCCGCATAGTAGTTACCGTACTGAATATCCTTCGC |
| --- | --- |
| **scFv_Y105A_** | Fw:CGGTAACGCGTATTACGGCATGGACGTTTGGGGC  Rv:CGTAATACGCGTTACCGTACTGAATATCCTTCGCGCAATAGTACA |
| **scFv_D99N_** | Fw:CGCGAAGAACATTCAGTACGGTAACTACTATTACGGCATGG  Rv:CTGAATGTTCTTCGCGCAATAGTACAGCGCGGTGTCT |
| **scFv_R228E_** | Fw:CCAGCAAGAAAGCAACTGGCCGATCACCTTTGGTC  Rv:AGTTGCTTTCTTGCTGGCAGTAATAAACCGCGAAATCTTCC |
| **scFv_Y169A_** | Fw:CAGCGCGCTGGCGTGGTATCAGCAAAAGCC  Rv:CCAGCGCGCTGCTAACGCTCTGGCTCGC |

**Figure S4. RMSD profiles of the Cα atoms of VH+VL, VH, VL and CD20 structures for all the type systems.** A) scFv_wild_/CD20, B) scFv_D99N_/CD20, C) scFv_Y105A_/CD20, D) scFv_Y107A_/CD20, E) scFv_Y169A_/CD20, F) scFv_R228E_/CD20, system. The structures obtained during the MD simulations were compared to their respective initial reference structures, where the reference structure for all variants scFv_type_/CD20 systems corresponded to the final configuration of the scFv_wild_/CD20 complex obtained at 700 ns. Simulations were conducted for 700 ns for the scFv_wild_/CD20 system and 200 ns for the scFv_type_/CD20 systems. Note that the x- and y-axis ranges differ for the scFv_wild_/CD20 system (panel A) due to its longer simulation time, which served as the reference trajectory for generating the variant systems. The scFv linker was disregarded from the RMSD analysis in both systems, as it presents flexible residues of glycine and serine, and presents high RMSD fluctuations. The scFv without the linker was denoted as the VH+VL Additionally, for the scFv_wild_/CD20 system (panel A), the N-terminal region of CD20 was excluded from the RMSD calculation due to its pronounced mobility, as illustrated in **Figure S5**. This exclusion reduces the overall displacement amplitude observed in panel A compared to analyses where the N-terminal motion is included (see **Figure S5**).

**Figure S5. RMSD profiles of the VH domain N-terminal region and of the VH and VH+VL domains excluding this region, and RMSD profile of VH+VL domain movements relative to the CD20 receptor.**  A) RMSD of the N-terminal region (black) fitted to the VH-Nterminal structure, RMSD and fitting of the VH-Nterminal (red), and RMSD and fitting of the VH-Nterminal+VL domains (blue). The N-terminal region exhibits significant conformational fluctuations. When this region was excluded from the VH domain during the RMSD analysis, both the VH and VH+VL domains showed lower RMSD fluctuations (red and blue, respectivelly) compared to the values observed in Figure S3A. The oscillation presented for the N-terminal (^1^EVQLVESGGGLVQ^13^) in the range of 450 to 600ns proves that the VH oscillation in the scF_wild_ system (Figure S3A) was caused by the N-terminal movement. B) RMSD of the VH-Nterminal+VL domains relative to the CD20 receptor, illustrating the movement of these domains throughout the simulation. During the first 200 ns, the scFv progressively improved its molecular fit to CD20. Thus, 300 ns was selected as the starting point for calculating the IIP and ΔG averages, representing a period of more stabilized interaction. The VH domain, excluding the N-terminal region, is referred here as VH-Nterminal. The combined VH and VL domains, excluding the VH N-terminal region, are referred here as VH-Nterminal+VL. The average RMSD and standard deviation calculated from 300 ns onward for the curve in graph B is 1.15 ± 0.16 nm.

**Table S4. Average RMSD values and standard deviations for the VH+VL, VH, VL, and CD20 structures in all complex systems, as well as for the VH+VL, VH, VL, and CDR-H3 RMSD in the isolated scFvs.** The CDR-H3 (residues 99-108) RMSD was calculated after structural fitting using the five residues preceding (94-98) and five residues following (109-113) the loop. Average RMSD values were calculated, and the equilibrium time (teq = 100 ns) was considered. The averages and deviations presented correspond to the RMSD profiles shown in Figure S4, S5 and S7A-F. ((ver quanto foi considerado apra Wild type, visto que sao 700 ns de DM))

| **Systems** | **RMSD**  **Mean ± Deviation (nm)** | | | | | | |
| --- | --- | --- | --- | --- | --- | --- | --- |
|  | **VH+VL** | | **VH** | **VL** | | **CD20** | |
| **ScFv_wild_/**  **CD20** | 0.28 ± 0.01* | 0.29 ± 0.02* | | | 0.24 ± 0.01 | | 0.32 ± 0.01 |
| **ScFv_D99N_/**  **CD20** | 0.17 ± 0.02 | 0.16 ± 0.02 | | | 0.14 ± 0.01 | | 0.15 ± 0.02 |
| **scFv_Y105A_/CD20** | 0.21 ± 0.01 | 0.15 ± 0.02 | | | 0.24 ± 0.03 | | 0.14 ± 0.02 |
| **scFv_Y107A_/CD20** | 0.16 ± 0.01 | 0.13 ± 0.01 | | | 0.15 ± 0.02 | | 0.13 ± 0.01 |
| **scFv_Y169A_/CD20** | 0.17 ± 0.01 | 0.12 ± 0.01 | | | 0.19 ± 0.02 | | 0.13 ± 0.01 |
| **scFv_R228E_/CD20** | 0.17 ± 0.02 | 0.18 ± 0.01 | | | 0.10 ± 0.02 | | 0.15 ± 0.01 |
|  | **VH+VL** | **VH** | | | **VL** | | **RMSD CDR-H3** |
| **scFv_wild_** | 0.30 ± 0.01 | 0.27 ± 0.01 | | | 0.26 ± 0.01 | | 0.27 ± 0.04 |
| **scFv_D99N_** | 0.29 ± 0.02 | 0.25 ± 0.02 | | | 0.17 ± 0.01 | | 0.57 ± 0.11 |
| **scFv_Y105A_** | 0.22 ± 0.01 | 0.20 ± 0.01 | | | 0.19 ± 0.02 | | 0.37 ± 0.06 |
| **scFv_Y107A_** | 0.20 ± 0.02 | 0.22 ± 0.03 | | | 0.12 ± 0.01 | | 0.30 ± 0.08 |
| **scFv_Y169A_** | 0.18 ± 0.02 | 0.19 ± 0.01 | | | 0.13 ± 0.01 | | 0.18 ± 0.06 |
| **scFv_R228E_** | 0.30 ± 0.03 | 0.31 ± 0.03 | | | 0.16 ± 0.03 | | 0.44 ± 0.12 |

*The average was calculated from the graph in Fig.S5A, excluding the N-terminal

**Figure S6. Structural representation of the scFv_wild_ and CD20 in different times of the MD simulation showing the VH N-terminal differences.** A) Structure collected at 80 ns of simulation. B) Structure collected at 400 ns of simulation. C) Structure collected at 464 ns of simulation. D) Structure collected at 700 ns of simulation. The scFv is shown as a gray cartoon, with the VH N-terminal highlighted in blue. The CD20 is shown as a green cartoon.


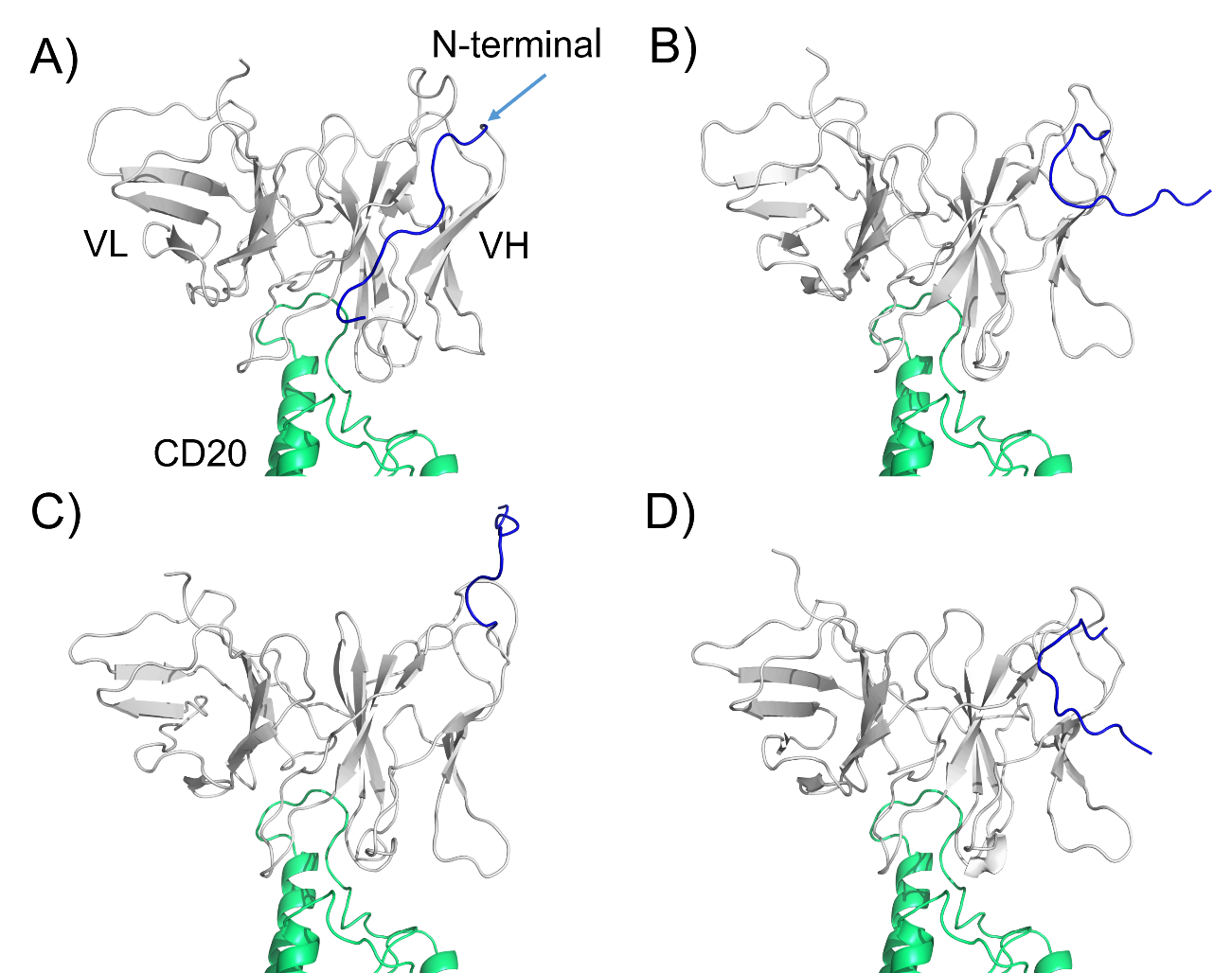


**Figure S7. Structural dynamics and conformational behavior of scFv variants in aqueous solution.**

(A–F) Root mean square deviation (RMSD) profiles of scFv wild and variants during 300 ns MD in water. RMSD values were calculated for the entire scFv (VH+VL, black), VH domain (green), VL domain (red), and the CDR-H3 loop (blue). The CDR-H3 RMSD was calculated after structural fitting using the five residues preceding and five residues following the loop. Specifically, residues 94–98 and 109–113 (Cα atoms) were used for alignment, while RMSD values correspond to the CDR-H3 region (residues 99–108). Panels correspond to scFv_wild_ (A), scFv_D99N_ (B), scFv_Y105A_ (C), scFv_Y107A_ (D), scFv_Y169A_ (E), and scFv_R228E_ (F).

(G–L) Structural representations shown were generated using the final configuration obtained from the MD trajectory of the scFv variants system in aqueous solution (300 ns). Representative top-view conformations of scFv, outside the antigen-binding interface, illustrating the spatial organization of complementarity-determining regions (CDRs). Structures are displayed in cartoon representation, where framework regions are colored light blue and remaining CDRs are shown in gray. The CDR-H3 loop, containing Asp99, Tyr105, and Tyr107, is highlighted in blue, while CDR-L1 (Tyr169) is highlighted in green and CDR-L3 (Arg228) is highlighted in red. Key side chains are represented as orange sticks. The residue corresponding to each mutation site is indicated by a red dashed circle in the respective panels.

These structural representations illustrate the conformational effects induced by mutations relative to the wild-type scFv. The wild-type structure (A and G) shows moderate positional fluctuations of CDR-H3 compared to the variants. Charge-altering mutations in scFv_D99N_ and scFv_R228E_ (B, F, H, and L) promote increased conformational instability of CDR-H3. In contrast, variants scFv_Y107A_ and scFv_Y169A_ (D, E, J, and K) show spatial proximity between Arg228 and Asp99, favoring the formation of a salt bridge (J and K), which induces torsional displacement of CDR-H3. In the wild-type and Y105A variant (C and I), Asp99 contributes to stabilizing the internal region of CDR-H3, maintaining a conserved loop orientation similar to that observed in the scFv_wild_ reference structure.

Salt bridge formation was evaluated by calculating the minimum distance between Arg228 and Asp99 residues using the gmx mindist tool. Distances were computed for all systems after 100 ns of MD simulations. The average distances (mean ± standard deviation) were 0.85 ± 0.15 nm for scFv_Wild_, 0.55 ± 0.12 nm for scFv_D99N_, 0.79 ± 0.09 nm for scFv_Y105A_, 0.30 ± 0.17 nm for scFv_Y107A_, 0.42 ± 0.25 nm for scFv_Y169A_, and 0.77 ± 0.11 nm for scFv_R228E_. The distances shown in panels J and K are displayed in angstroms (Å). Cation–π interactions were further evaluated by measuring the distance between the guanidinium group of Arg228 and the aromatic rings of Tyr107 and Tyr169. The Tyr107–Arg228 distance averaged 0.47 ± 0.09 nm in the scFv_wild_ system, consistent with a stabilizing interaction, whereas the Tyr169–Arg228 distance increased to 0.63 ± 0.15 nm in the Y169A variant, suggesting a weaker and more transient interaction.


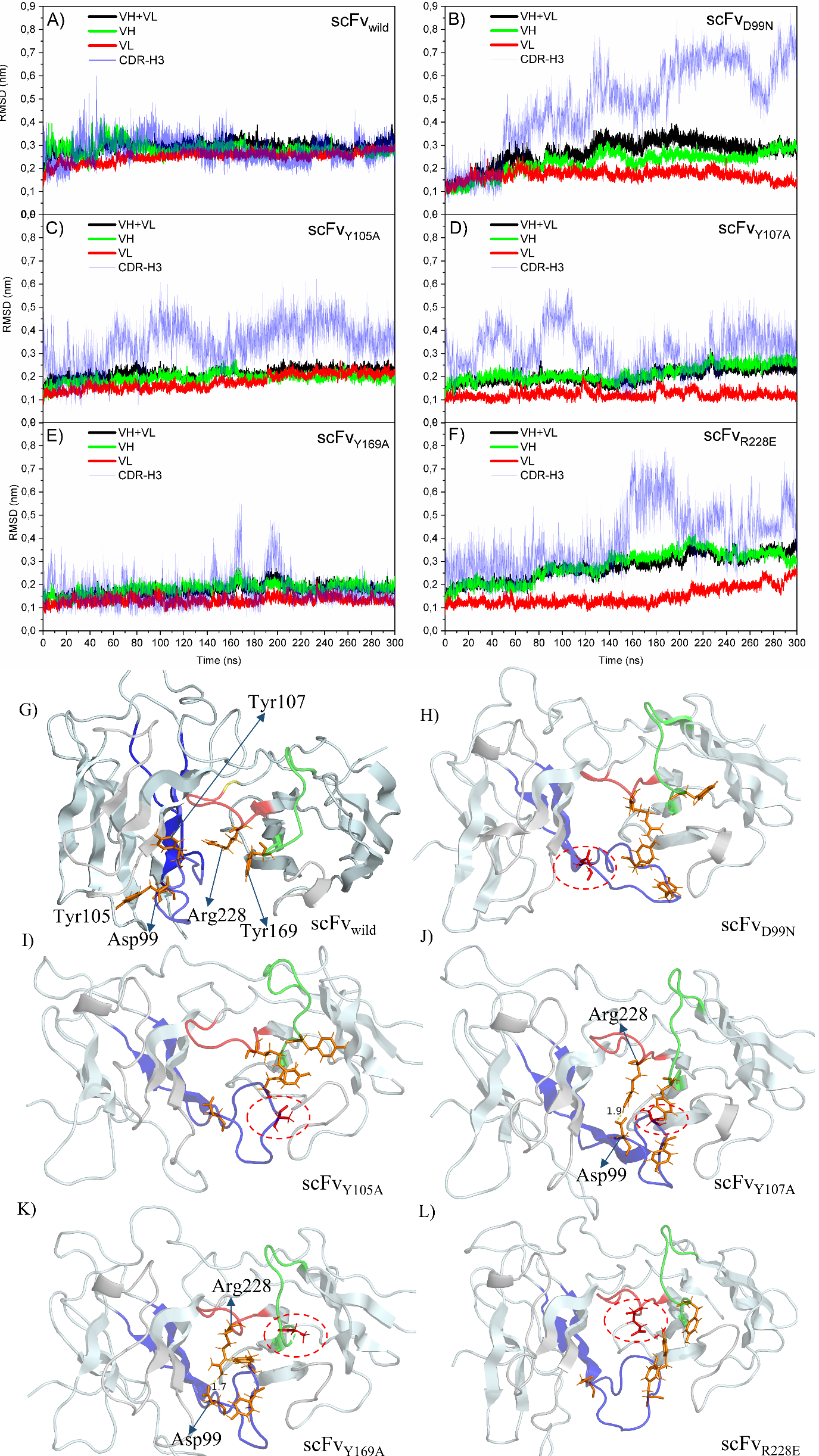


**Figure S8. IIP profiling between scFvs and CD20.** IIP plots calculated between scFvs and CD20 in all systems. A) scFv_wild_/CD20 (700 ns), B) scFv_D99N_/CD20 (200 ns), C) scFv_Y105A_/CD20 (200 ns), D) scFv_Y107A_/CD20 (200 ns), E) scFv_Y169A_/CD20 (200 ns), F) scFv_R228E_/CD20 (200 ns). Note that the scFv_wild_/CD20 system (panel A) was simulated for a longer time (700 ns), serving as the reference trajectory for generating the variant systems. The cutoff radius for the interaction potential is 1.3 nm*.* Mean and standard deviation values of IIP were calculated from teq = 100 ns and are indicated in parentheses within each graph.





**Figure S9. Structural representation of the interface between scFv_wild_ and CD20 at different times of the MD simulation, shown in three views PCA analyses: frontal, lateral, and posterior.** A, B, C) Initial structure of the simulation. D, E, F) Final structure after 700 ns of simulation. The scFv is shown as a green cartoon, highlighting key residues from the VH and VL regions involved in the interaction interface, represented as magenta sticks. The VH residues are: Tyr32, Asp99, Asn104, Tyr105 and Tyr107. The VL residues are: Tyr169, Asp187 and Arg228. The CD20 is shown as a gray cartoon, with the two loops containing the key interacting residues highlighted in blue cartoon and the residues shown as orange sticks. The CD20 residues are: Lys108, Pro132, Lys135, Tyr144 and Glu147. View 1: front view; View 2: rotated 90 degrees; View 3: rotated 180 degrees. G, H) PCA analyses for the scFv_wild_/CD20 complex. G) PC1 and PC2 components plotted as a function of time over 700 ns of simulation. H) Projection of PC1 vs. PC2. The PCA was performed using CD20 as the reference structure, excluding the N-terminal and the linker region between the VH and VL domains of the scFv.

**
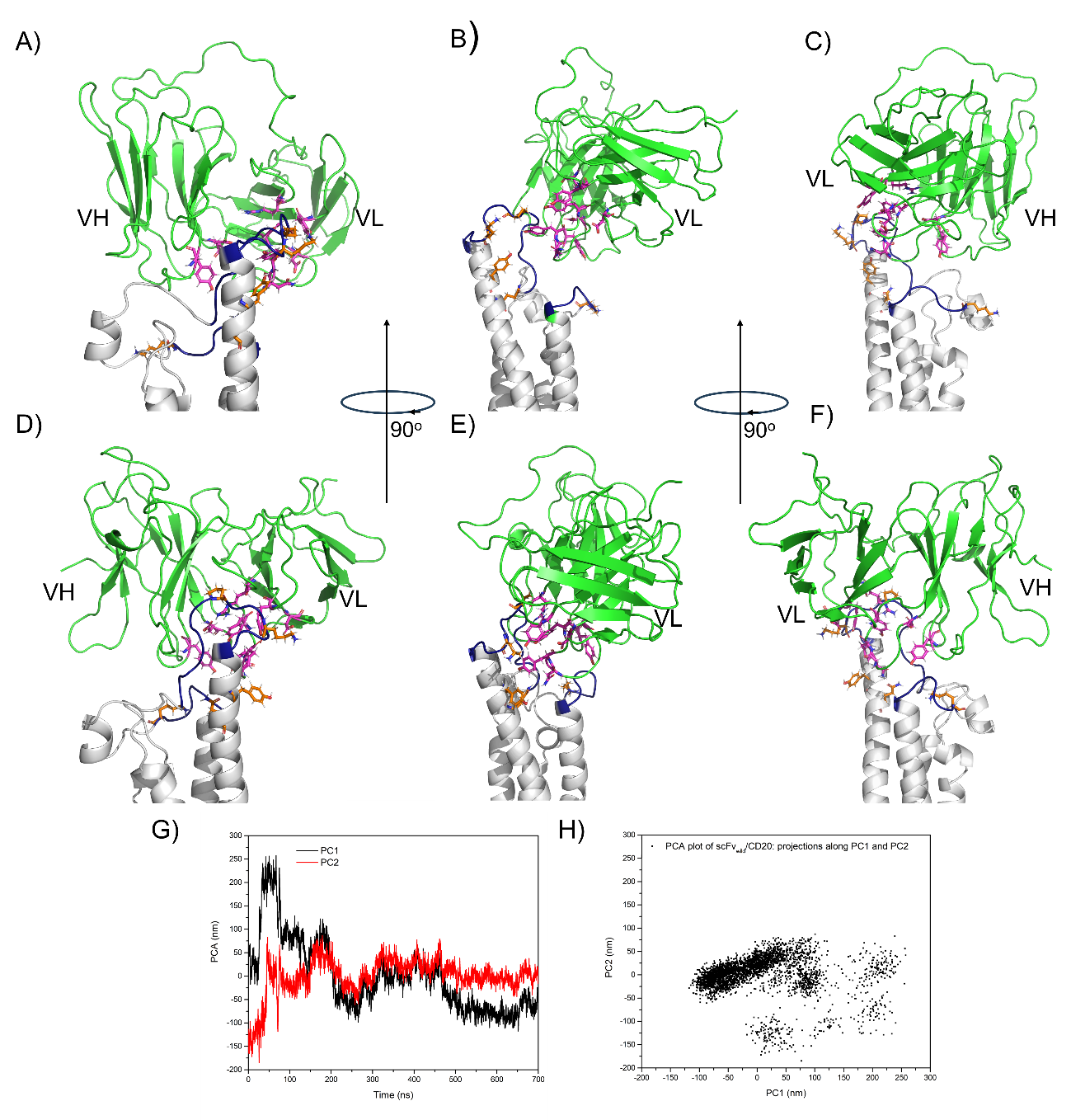
**

**Figure S10.** **ΔG_Binding_ curves of the complexes scFvs/CD20 over the simulation.** A) scFv_wild_/CD20 (700 ns), B) scFv_D99N_/CD20 (200 ns), C) scFv_Y105A_/CD20 (200 ns), D) scFv_Y107A_/CD20 (200 ns), E) scFv_Y169A_/CD20 (200 ns), F) scFv_R228E_/CD20 (200 ns)


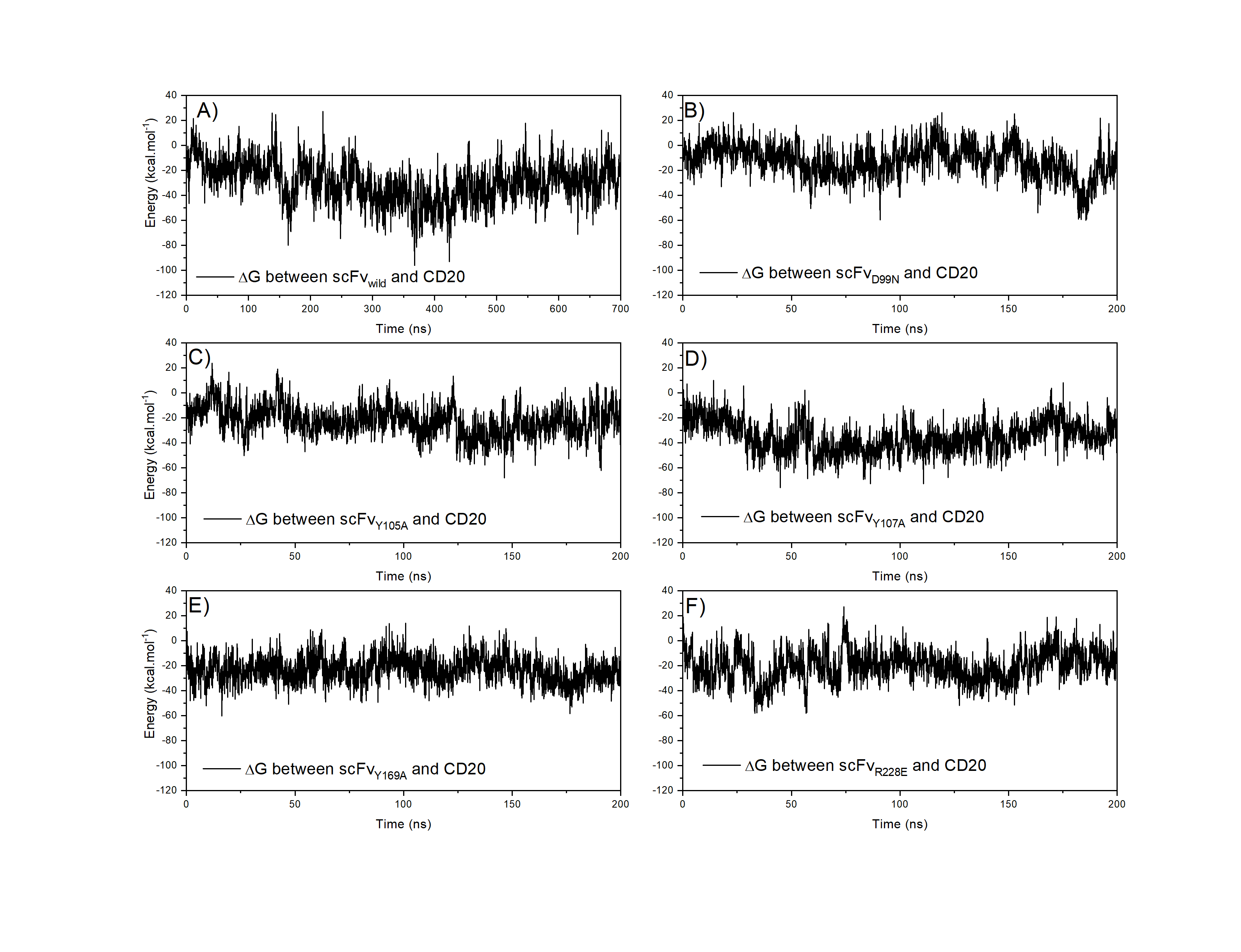


**Table S5. Summary of the average ΔG_binding_ values obtained by MM/PBSA for the scFv/CD20 systems.** Average ΔG_binding_ values were calculated, and the equilibrium time (teq) of 300 ns for scFv_wild_/CD20 system and 100 ns for scFv_type_/CD20 systems were considered. The averages and deviations presented correspond to the ΔG_binding_ profiles shown in Figure S9.

| **Systems** | **ΔG_Binding_**  **(kcal mol^-1^)** |
| --- | --- |
| **scFv_wild_/CD20** | −34.5 ± 15.6 |
| **scFv_D99N_/CD20** | −13.4 ± 14.5 |
| **scFv_Y105A_/CD20** | −26.2 ± 12.1 |
| **scFv_Y107A_/CD20** | −34.4 ± 10.9 |
| **scFv_Y169A_/CD20** | −24.0 ± 10.6 |
| **scFv_R228E_/CD20** | −19.1 ± 11.3 |

**Figure S11. Per-residue binding free energy (ΔG_res) and relative differences (ΔΔG_res) of scFv residues interacting with CD20.** (A) Absolute ΔG_res distribution for the scFv_wild_/CD20 system. (B–F) Per-residue binding free energy differences (ΔΔG_res) between each variant and the wild-type complex. Positive ΔΔG_res values indicate less favorable binding, whereas negative values indicate more favorable binding. Key paratope residues with relevant variations include Tyr32 (CDR-H1), Asn54 and Ser55 (CDR-H2), Tyr102, Asn104, Tyr105 and Tyr107 (CDR-H3), Tyr169 (CDR-L1), Asp187 (CDR-L2), and Arg228 and Ser229 (CDR-L3). (A) scFv_wild_/CD20, (B) scFv_D99N_/CD20, (C) scFv_Y105A_/CD20, (D) scFv_Y107A_/CD20, (E) scFv_Y169A_/CD20, (F) scFv_R228E_/CD20.

**Figure S12. Per-residue binding free energy (ΔG_res) and relative differences (ΔΔG_res) of CD20 residues in interaction with scFvs.** (A) Absolute ΔG_res distribution for the scFv_wild_/CD20 system. (B–F) Per-residue binding free energy differences (ΔΔG_res) between each variant and the wild-type system. Positive ΔΔG_res values indicate less favorable binding compared with the wild type, whereas negative values indicate more favorable binding. Key epitope residues showing relevant variations include His105, Lys108, Asn126, Glu128, Pro129, Ala130, Pro132, Lys135, Tyr144, and Gln147. (A) scFv_wild_/CD20, (B) scFv_D99N_/CD20, (C) scFv_Y105A_/CD20, (D) scFv_Y107A_/CD20, (E) scFv_Y169A_/CD20, (F) scFv_R228E_/CD20.

**Table S6. Mapping of hydrogen-bond interactions between scFv and CD20 residues.**

Hydrogen-bond (HB) occurrence was calculated using the gmx hbond program in GROMACS, applying a distance-based criterion. Only interactions present in more than 10% of the trajectory are reported. The first column lists the scFv residues, while subsequent columns indicate the percentage of HB occurrence with each CD20 residue or phospholipid (PL). When percentages are shown in pairs (e.g., 50%, 50%), they correspond respectively to hydrogen bonds involving the backbone carbonyl and amine groups of the same residue. This representation highlights the main contact residues that define the scFv/CD20 interface.


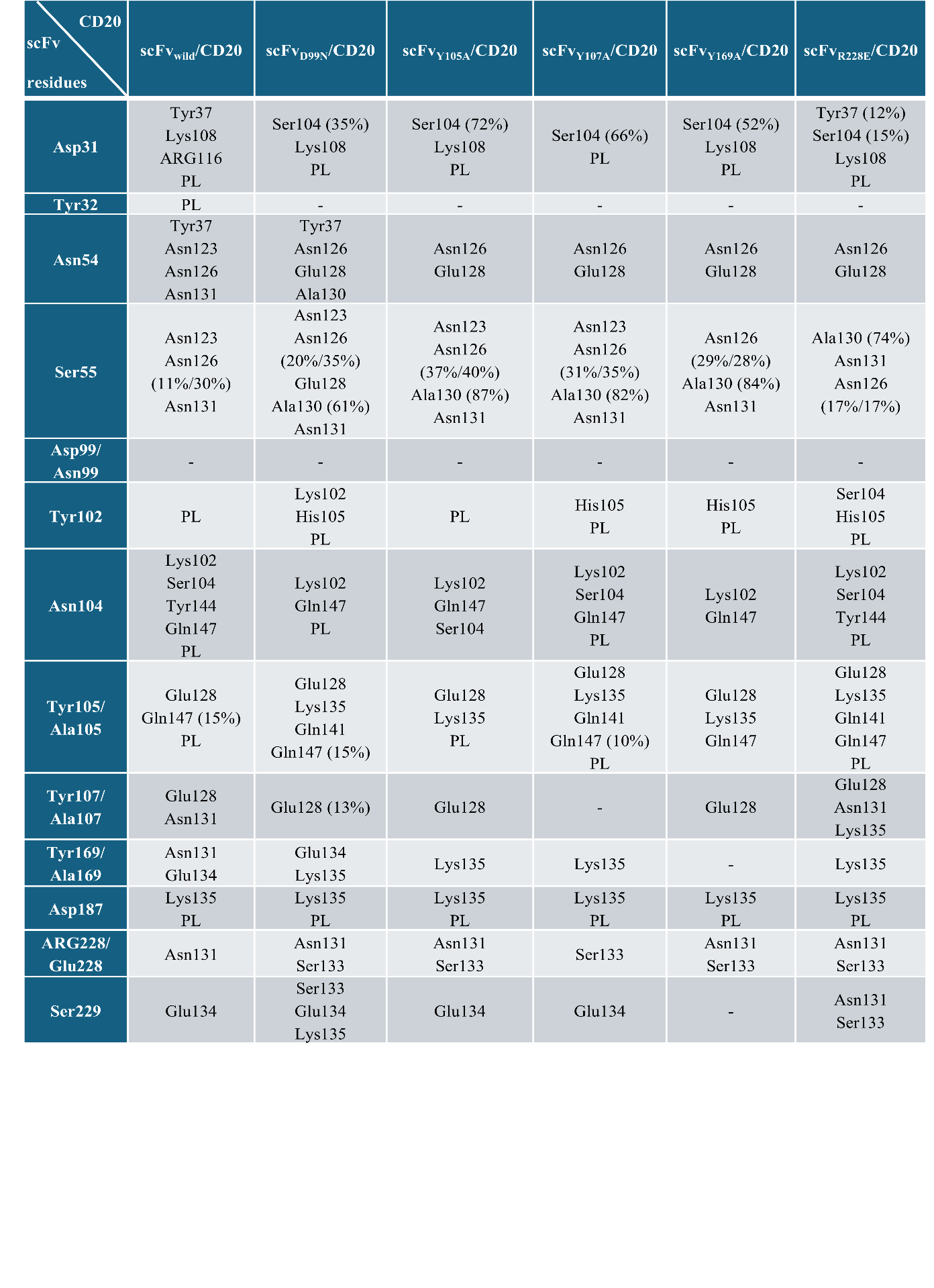


**Table S7. Mapping of hydrogen-bond interactions between CD20 and scFv residues.** Hydrogen-bond (HB) occurrence was calculated using the gmx hbond program in GROMACS, applying a distance-based criterion. Only interactions present in more than 10% of the trajectory are reported. The first column lists the scFv residues, while subsequent columns indicate the percentage of HB occurrence with each CD20 residue or phospholipid (PL). When percentages are shown in pairs (e.g., 50%, 50%), they correspond respectively to hydrogen bonds involving the backbone carbonyl and amine groups of the same residue. This representation highlights the main contact residues that define the scFv/CD20 interface.


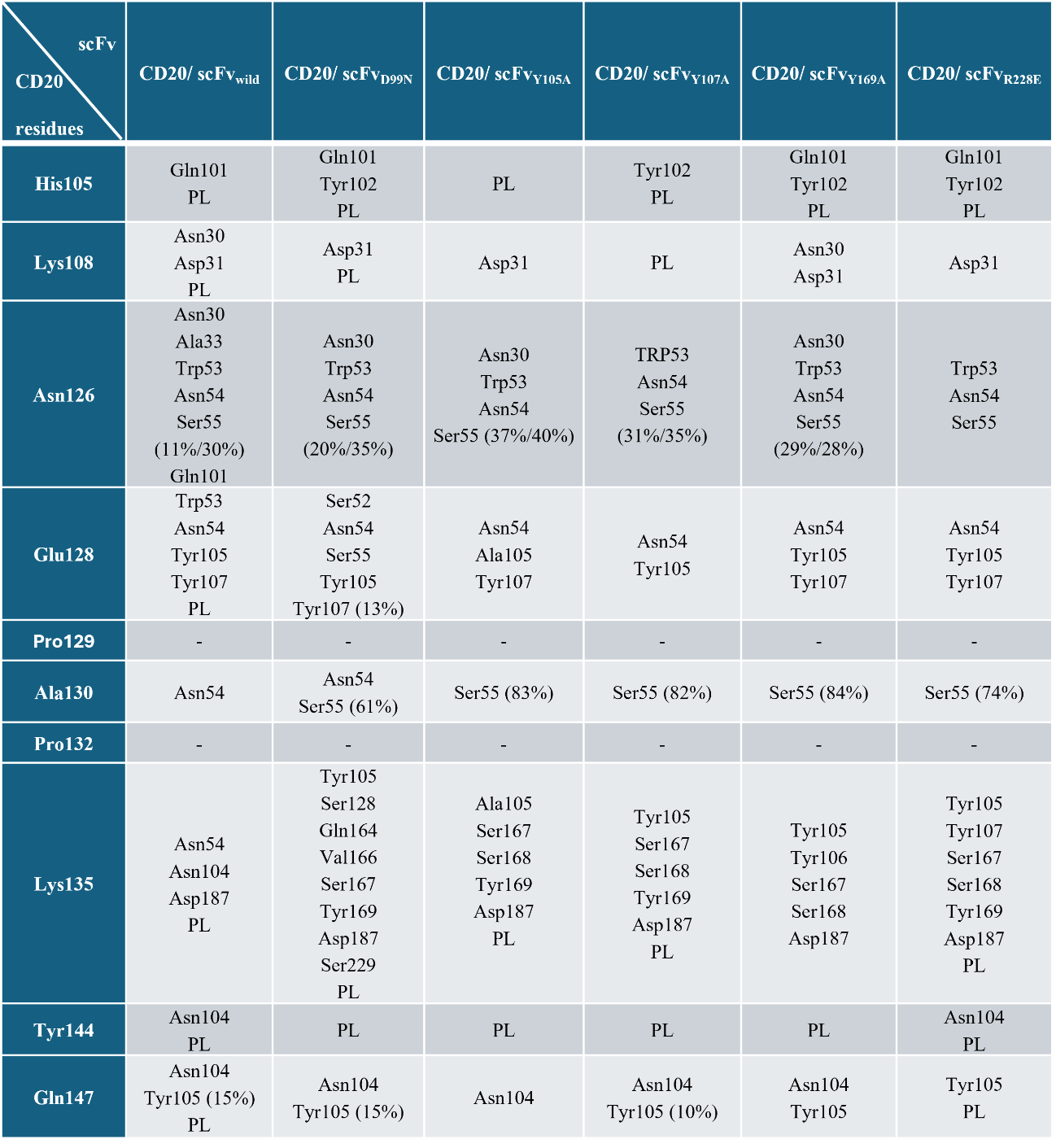


**Figure S13. SDS-PAGE analysis of total protein extracts from aliquots T_0_ and T_16_.**

Lane MW: molecular weight marker (True Color High Range, Sinapse); Lane 1: protein profile of aliquot T0; Lane 2: protein profile of aliquot T16. The scFv_wild_^-^SUMO-tag is expected to be approximately 41 kDa. kDa: kilodaltons.

**
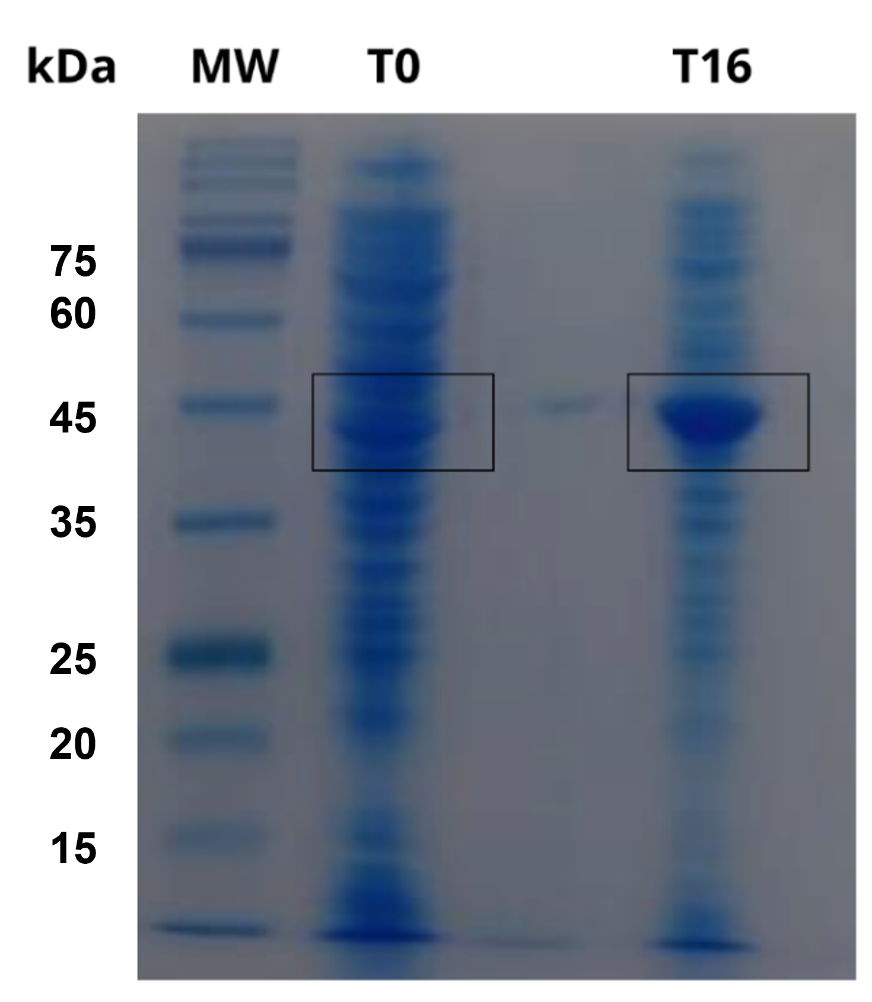
**

**Figure S14. SDS-PAGE and Western blot analysis of scFv_wild_ expression and purification.** (A) SDS-PAGE analysis of Ni-NTA affinity chromatography fractions from scFv_wild_ expression. Lane MW: molecular weight marker (True Color High Range, Sinapse). Lanes F1-F7: eluted fractions, showing a band at ~41 kDa corresponding to scFv_wild_-SUMO, with higher intensity in fractions F4-F7 (highlighted). (B) Western blot analysis of the same eluted fractions (F4-F7). The Western blot was performed using an anti-polyhistidine alkaline phosphatase–conjugated mouse monoclonal antibody (Sigma) at a 1:5000 dilution and detected using the Western Blue Stabilized Substrate for Alkaline Phosphatase (Promega). Lane MW: molecular weight marker. Lanes F5–F7 show a positive signal at ~41 kDa, confirming protein expression. (C) SDS-PAGE analysis of scFv_wild_ after size-exclusion chromatography performed using the Superdex 200 Increase 10/300 GL column (Cytiva). Lane MW: molecular weight marker. Lane 2: fraction displaying a prominent band at ~41 kDa. (D) SDS-PAGE analysis of reverse affinity chromatography using the HisLink Protein Purification Resin (Promega). Lane MW: molecular weight marker. Lane 1: SUMO fusion protein. Lane 2: scFv_wild_ after SUMO cleavage, observed at ~26 kDa. kDa: kilodaltons.


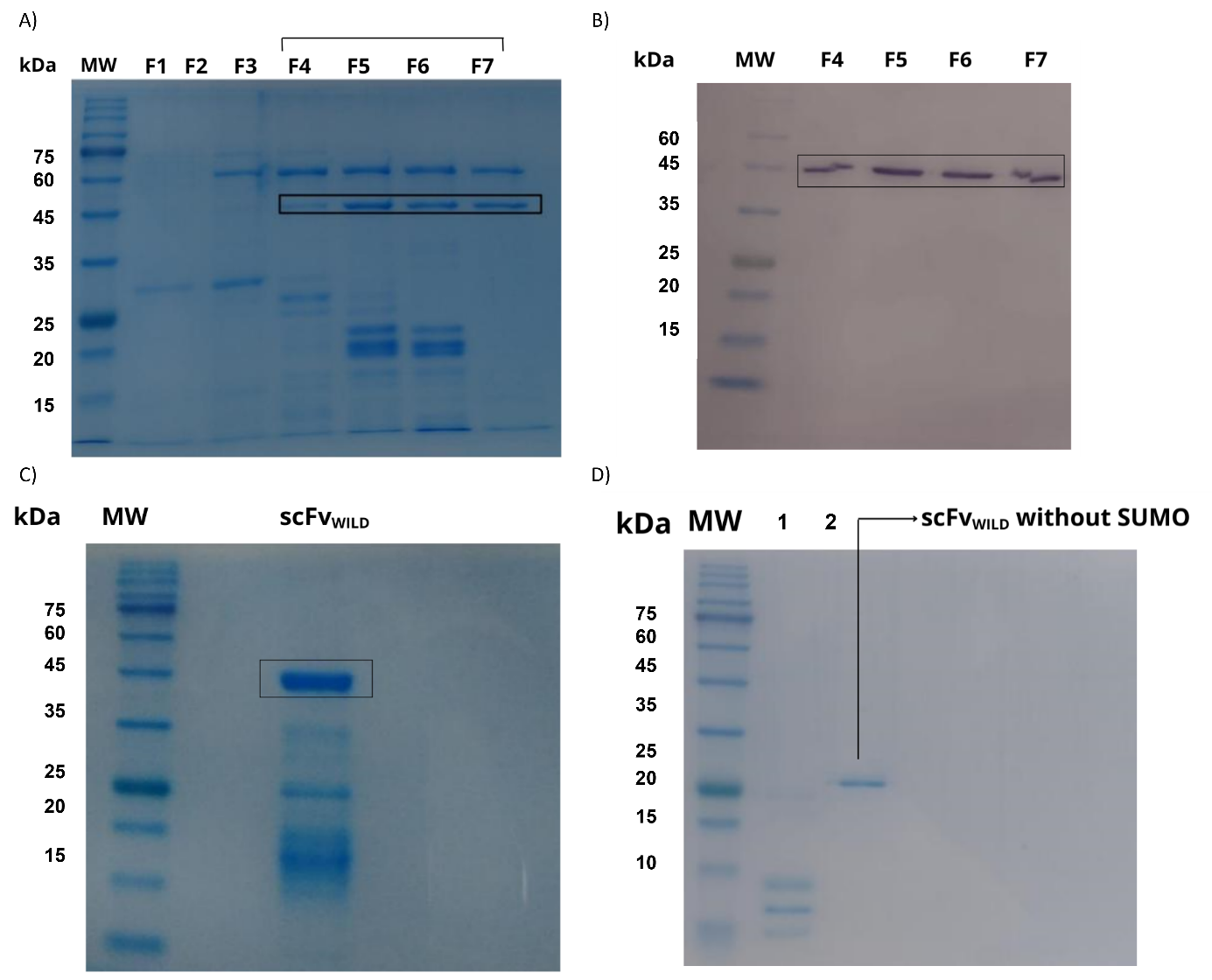


**Figure S15. SDS-PAGE analysis of scFv expression at T0 and T16.** Total protein samples of scFv_wild_, scFv_Y107A_, scFv_D99N_, scFv_R228E_, scFv_Y169A_, and scFv_Y105A_ (~41 kDa) were resolved by SDS-PAGE. Gel 1 - Lane MW: molecular weight marker (True Color High Range, Sinapse). Lane 1: scFv_wild_ (T16); Lanes 2–3: scFv_Y107A_ (T0, T16). Lanes 4–5: scFv_D99N_ (T0, T16). Lanes 6–7: scFv_R228E_ (T0, T16). Lanes 8–9: scFv_Y169A_ (T0, T16). Gel 2 - Lanes 1–2: scFv_wild_ (T0, T16). Lanes 3–4: scFv_Y105A_ (T0, T16). kDa, kilodaltons.


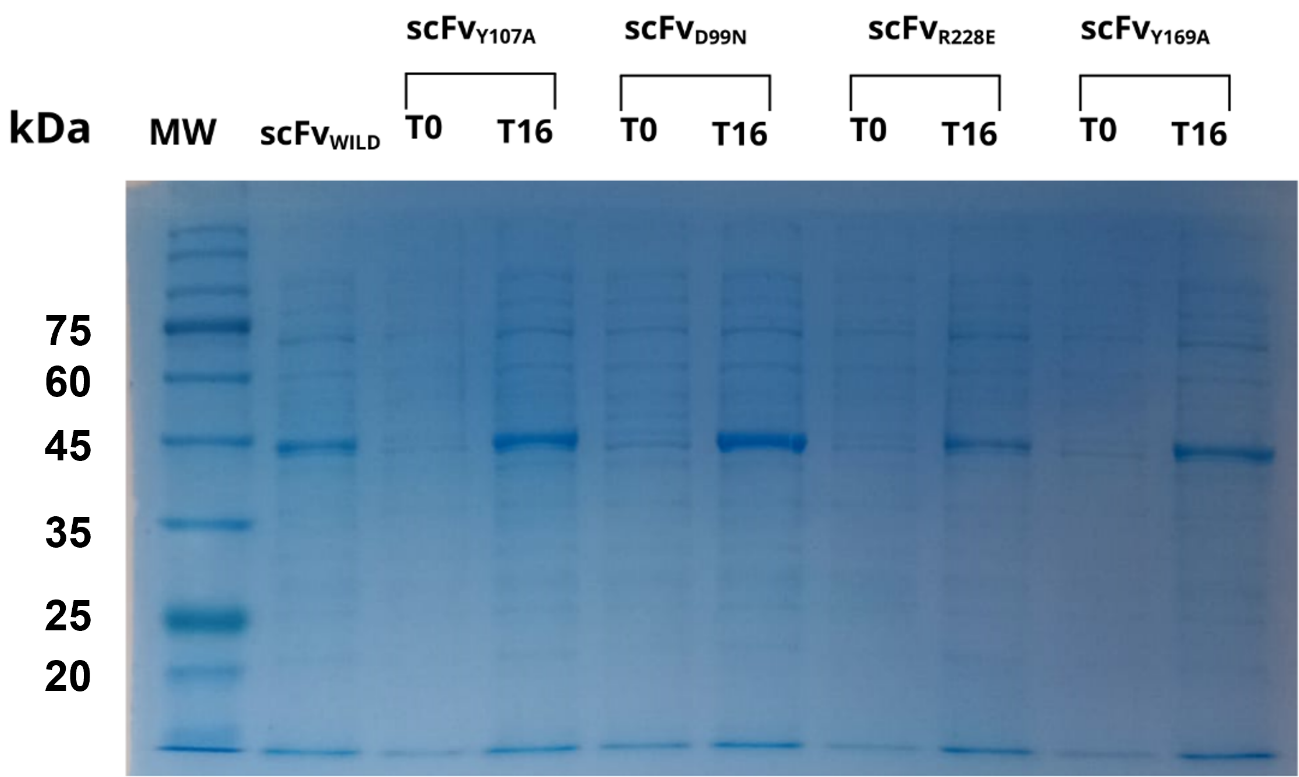


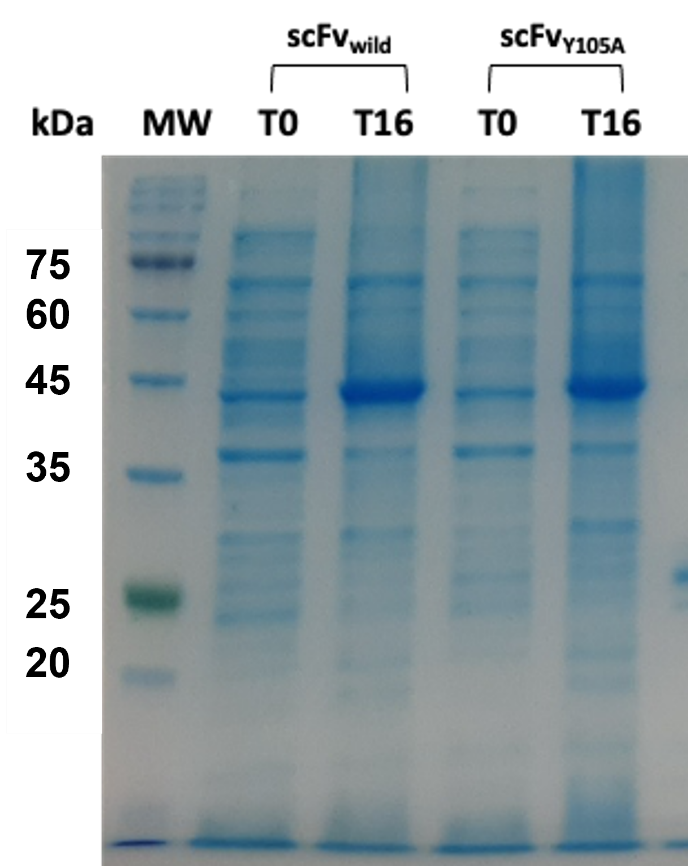


**Figure S16. SDS-PAGE and Western blot analysis of scFv_wild_ and variants after SUMO cleavage.** (A) SDS-PAGE analysis of reverse affinity chromatography fractions containing scFv_wild_ and variants. Proteins were analyzed after SUMO removal, resulting in a molecular weight of ~26 kDa. Lane MW: molecular weight marker (True Color High Range, Sinapse). Lane 1: scFv_wild_. Lane 2: scFvY107A. Lane 3: scFvD99N. Lane 4: scFvY105A. Lane 5: scFvR228E. Lane 6: scFvY169A. (B) Western blot analysis of the same samples using an anti-HA tag monoclonal antibody conjugated to HRP (Invitrogen) at a 1:5000 dilution, with detection performed using the Pierce ECL Western Blotting Substrate (Thermo Fisher Scientific). All scFvs were detected at ~26 kDa. kDa: kilodaltons.


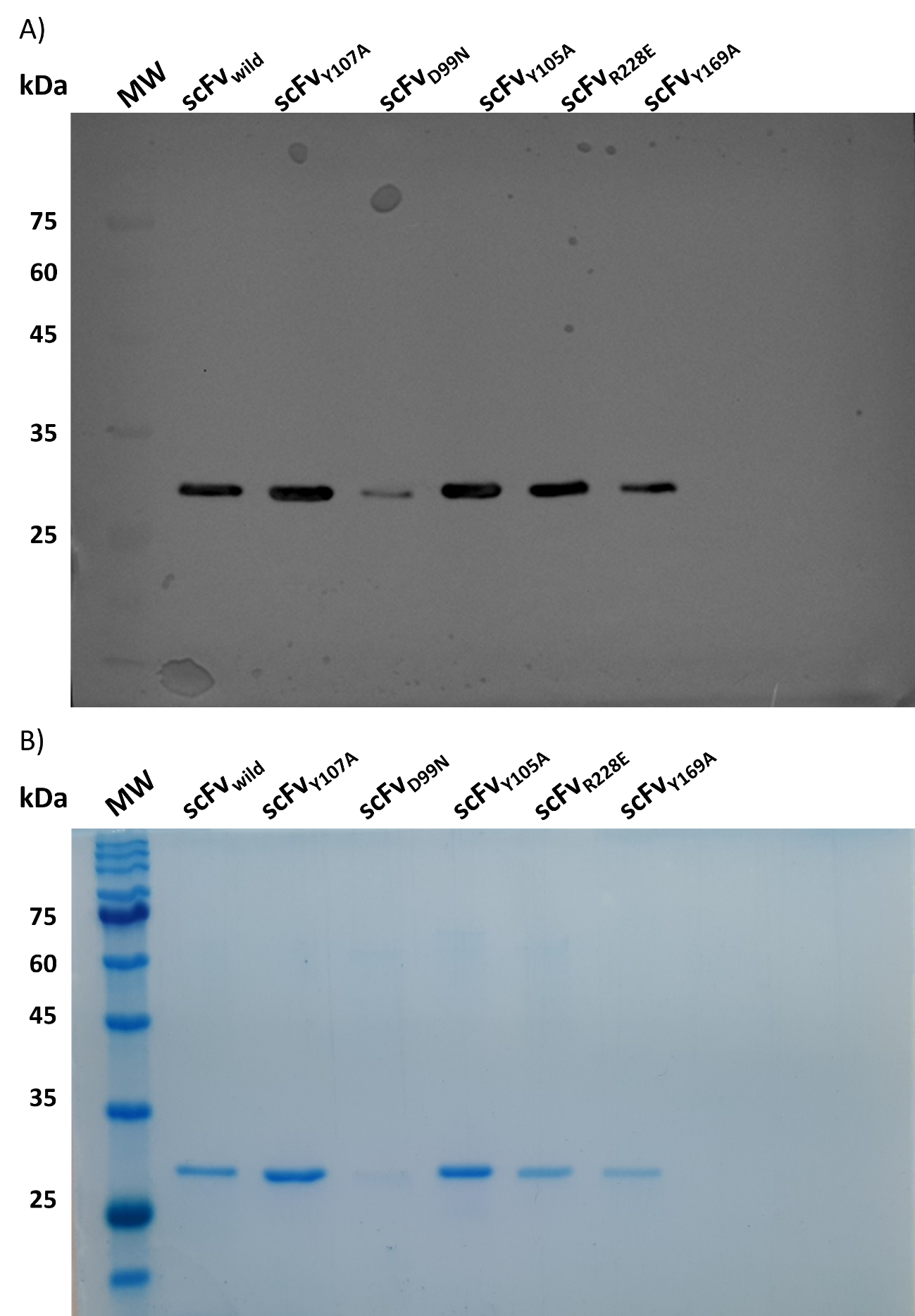


**Figure S17. Flow cytometry gating strategy for the selection of cell populations (Jurkat, CD20⁻; Raji, CD20⁺).** A) Singlet populations, corresponding to individual cells detected by the laser. B) Gating of the target cell population within the singlets. C) Control for nonspecific binding and lack of staining by the wild scFv in Jurkat cells (FITC channel). D) Control for nonspecific binding and CD20 antigen staining by the wild Ofatumumab scFv in Raji cells (FITC channel), showing the expected negative and positive staining patterns. WILD: wild Ofatumumab scFv; NSC: non-specific control.

**
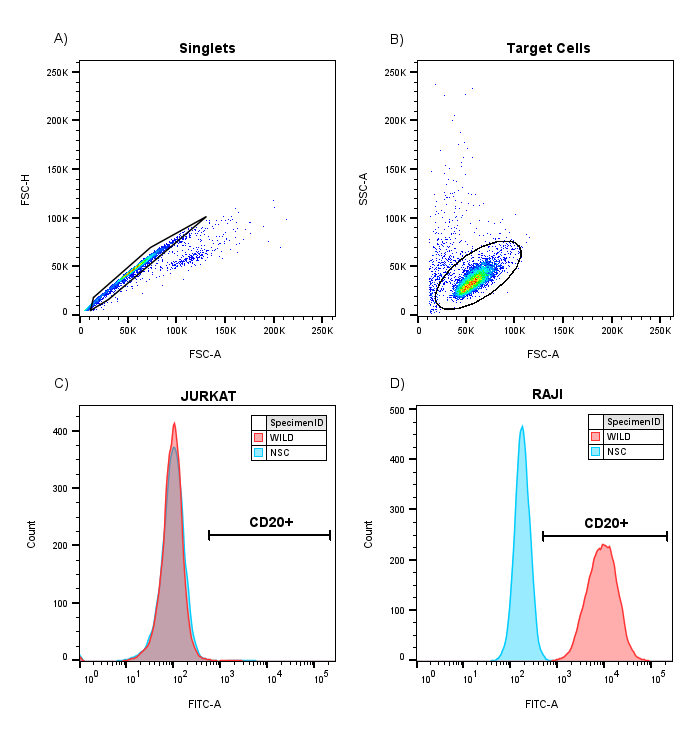
**

**Figure S18. Flow cytometry analysis of scFv binding controls.** (A) Jurkat cells (CD20⁻) incubated with a commercial anti-CD20 monoclonal antibody, showing no detectable fluorescence (negative control). (B) Raji cells (CD20⁺) incubated with the same antibody, confirming CD20 expression through positive fluorescence staining.


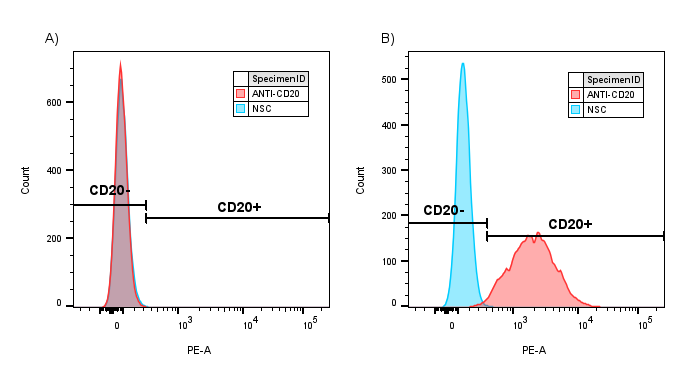


**Figure S19. Interactions between CD20 ECL1 and the Ofatumumab scFv, and intramolecular contacts within the scFv stabilizing the paratope, during molecular dynamics simulations.** A) plot of the minimum distance between residues of the Ofatumumab epitope on CD20 ECL1 (Ile36 and Tyr37, shown in bold), as described by Du et al. (2009) and Teeling et al. (2006)[3,5] and residues of the scFv (Trp53 and Asn30). Distances were computed using the gmx mindist tool for scFv_wild_/CD20 system (700ns), representing the smallest interatomic separation between the two residue sets throughout the simulation. B) Structural representation of the same region corresponding to panel A. CD20 residues are shown as sticks with gray carbon atoms, while scFv residues are shown in orange. The smaller CD20 loop (ECL1, containing the interaction region proposed by Du et al.) is shown in light gray and is surrounded by the larger ECL2 loop, whose subregions ECL2a and ECL2b are colored in red and blue, respectively. C) Minimum-distance plot between Asp99 (scFv) and Tyr107 (scFv), showing a stable intradomain interaction that contributes to the local stabilization of the CDR-H3 loop. D) Minimum-distance plot between Asp99 and Arg228, revealing a long-range electrostatic coupling between the heavy and light chains that supports the conformational arrangement of the tyrosine cluster. (E–F) Two structural views of the tyrosine cluster (Tyr105, Tyr107, and Tyr169) together with Arg228 and Asp99, highlighting the hydrogen-bond network between the Asp99 carboxyl oxygen and the amine hydrogen of Tyr107, indicated by a yellow dashed line, which stabilizes the H3 loop conformation highlighted in light orange in the cartoon representation of the main chain. The distance between the Asp99 carboxyl group and the Arg228 guanidinium group is also shown as a yellow dashed line, illustrating how Asp99 helps to position and orient Tyr107 within the binding interface. Panels B, E, and F were generated from the final configuration obtained at the end of the MD simulation of scFv_wild_/CD20 (700 ns).

**
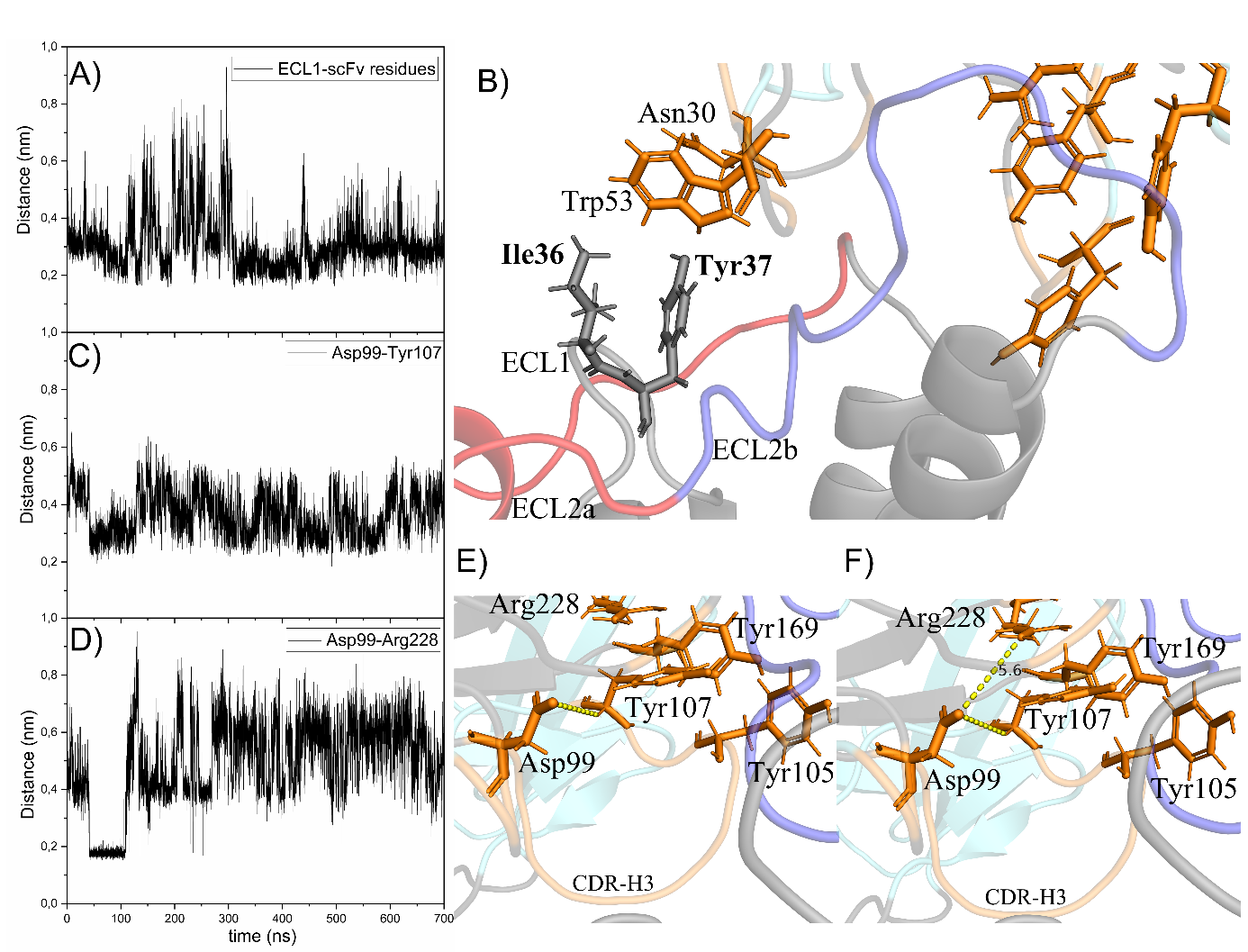
**

***Data and Software Availability***

The GitHub repository associated with this study is publicly available at https://github.com/marcos-lourenzoni-gepess/CSBJ.

The repository includes:

- Input coordinate and topology files for each CD20–scFv system (wild-type and mutants);
- GROMACS molecular dynamics parameter files (.mdp);
- MMPBSA input and output files used for ΔG_binding and ΔG_res calculations;
- Python analysis scripts for trajectory processing, minimum distance measurements, and residue interaction energy decomposition;
- Figures and plotting scripts used to generate all graphs presented in the manuscript;
- Sequence alignment data and structural models (PDB files) of CD20 and scFv variants.
- Raw flow cytometry data (Excel file) used for experimental binding validation.

No proprietary or confidential data were used in this study. Complete molecular dynamics trajectories (XTC and TPR files) are available from the corresponding author upon reasonable request.

**Software used:**

- GROMACS 2018.8 (for molecular dynamics simulations)
- gmx_MMPBSA v1.5.5 (for MM-PBSA and per-residue free-energy decomposition)
- Python 3.10 with NumPy, MDTraj, and Matplotlib (for trajectory analysis and visualization)
- PyMOL 2.5 with APBS plugin (for electrostatic potential and structural representation)
- Clustal Omega (for sequence alignment)
- ESPript 3.0 (for sequence alignment visualization)

All scripts and analysis workflows are fully documented within the GitHub repository to ensure transparency and reproducibility of all computational analyses and figures presented in this work.

Bottom of Form

References

1. Sievers F, Wilm A, Dineen D, Gibson TJ, Karplus K, Li W, et al. Fast, scalable generation of high-quality protein multiple sequence alignments using Clustal Omega. Mol Syst Biol [Internet]. 2011 Apr 16;7(539):1–6. Available from: http://msb.embopress.org/cgi/doi/10.1038/msb.2011.75

2. Robert X, Gouet P. Deciphering key features in protein structures with the new ENDscript server. Nucleic Acids Res [Internet]. 2014 Jul 1;42(W1):W320–4. Available from: http://academic.oup.com/nar/article/42/W1/W320/2435247/Deciphering-key-features-in-protein-structures

3. Du J, Yang H, Guo Y, Ding J. Structure of the Fab fragment of therapeutic antibody Ofatumumab provides insights into the recognition mechanism with CD20. Mol Immunol. 2009;46(11–12):2419–23.

4. Kumar A, Planchais C, Fronzes R, Mouquet H, Reyes N. Binding mechanisms of therapeutic antibodies to human CD20. Science (80- ) [Internet]. 2020 Aug 14;369(6505):793–9. Available from: https://www.science.org/doi/10.1126/science.abb8008

5. Teeling JL, Mackus WJM, Wiegman LJJM, van den Brakel JHN, Beers SA, French RR, et al. The Biological Activity of Human CD20 Monoclonal Antibodies Is Linked to Unique Epitopes on CD20. J Immunol. 2006;177(1):362–71.
